# MiR‐378a‐3p as a putative biomarker for hepatocellular carcinoma diagnosis and prognosis: Computational screening with experimental validation

**DOI:** 10.1002/ctm2.307

**Published:** 2021-02-14

**Authors:** Fuliang Qian, Jinghan Wang, Ying Wang, Qian Gao, Wenying Yan, Yuxin Lin, Li Shen, Yufeng Xie, Xiaoqing Jiang, Bairong Shen

**Affiliations:** ^1^ Center for Systems Biology Soochow University Suzhou China; ^2^ Department of the First Biliary Surgery, Shanghai Eastern Hepatobiliary Surgery Hospital Navy Military Medical University Shanghai China; ^3^ Department of Oncology The First Affiliated Hospital of Soochow University Suzhou China; ^4^ Institutes for Systems Genetics, Frontiers Science Center for Disease‐related Molecular Network, West China Hospital Sichuan University Chengdu China

**Keywords:** cancer genesis and progression, hepatocellular carcinoma (HCC), microRNA biomarker, network structure characterization, unique regulatory and influential power

## Abstract

**Background:**

Hepatocellular carcinoma (HCC) is a malignant disease with high morbidity and mortality, and the molecular mechanism for the genesis and progression is complex and heterogeneous. Biomarker discovery is crucial for the personalized and precision treatment of HCC. The accumulation of reported microRNA biomarkers makes it possible to combine computational identification with experimental validation to accelerate the discovery of novel biomarker.

**Results:**

In the present work, we applied a rational computer‐aided biomarker discovery model to screen for the HCC diagnosis biomarker. Two HCC‐associated networks were constructed based on the microRNA and mRNA expression profiles, and the potential microRNA biomarkers were identified based on their unique regulatory and influential power in the network. These putative biomarkers were then experimentally validated. One prominent example among these identified biomarkers is MiR‐378a‐3p: It was shown to independently regulate several important transcription factors such as PLAGL2 and β‐catenin, affecting the β‐catenin signaling. Such mechanism may indicate a potential tumor suppressor role of MiR‐378a‐3p and the impact of its abnormal expression on the cell growth and invasion of HCC.

**Conclusions:**

A bioinformatics model with network topological and functional characterization was successfully applied to the identification of HCC biomarkers. The predicted microRNA biomarkers were than validated with experiments using human HCC cell lines, model animal, and clinical specimens. The results confirmed the prediction by our proposed model that miR‐378a‐3p was a putative biomarker for diagnosis and prognosis of HCC.

## Graphical abstract

A rational computer‐aided biomarker discovery model has been successfully applied to screen microRNA biomarker for hepatocellular carcinoma based on their unique regulatory and influential power in the microRNA‐mRNA network.

MiR‐378a‐3p was identified and validated as a biomarker for HCC diagnosis and prognosis, it was shown independently regulated several important transcription factors including PLAGL2 and β‐catenin, then affected the β‐catenin signaling, which could be the potential mechanism for its function as a tumor suppressor and its abnormal expression could affect the cell growth and invasion of HCC

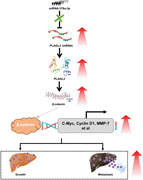

**Abbreviations**
AUC: area under the curve
CCK‐8: cell counting kit‐8
cDNA: complementary DNA
DEG: differentially expressed gene
FBS: fetal bovine serum
H&E: hematoxylin & eosin
HCC: hepatocellular carcinoma
HRP: horseradish peroxidase
IHC: immunohistochemistry
ISH: in situ hybridization
Luc: luciferase
NC: negative control
Neo: neomycin
NOD: novel out degree
PBS: phosphate‐buffered saline
POMA: Pipeline of Outlier MicroRNA Analysis
qPCR: quantitative polymerase chain reaction
ROC: receiver operating characteristic
RT: reverse transcription
s.c.: subcutaneous/subcutaneously
siRNA: small interfering RNA
SVM: support vector machine
TF: transcription factor
TFP: transcription factor percentage
TMA: tissue microarray
UTR: untranslated region

## 1 BACKGROUND

MicroRNAs are small noncoding RNAs with 22–24 nucleotides in length and important regulators in many biological systems. They regulate gene expression at the posttranscriptional level and can be detected in blood or tissues specifically and stably. Thus, using microRNAs as biomarkers holds potential for disease diagnosis, prognosis, and therapy.^1^

Nowadays, most microRNA biomarkers have been studied via biological and clinical experiments that focus on the functional role of individual microRNA, or its expression profiling to identify potential novel biomarkers. However, there are few researches applying theoretical network structures and calculation to investigate a large number of biomarkers.^2^, ^3^ The advent of “Big Data Era” provides opportunities and challenges for microRNA biomarker discovery from massive and diverse data. Bioinformatics and computer‐aided biomarker discovery have received increasing attention due to its feasibility, guidance, and effectiveness.

In the last decade, many bioinformatics algorithms or models have been developed for identifying microRNA biomarkers, which can be divided into three categories: mathematical models, machine learning, and network analysis. A mathematical model is a description of a model using various scoring functions and statistical methods to identify microRNA biomarkers. Based on the hypothesis that a microRNA is involved in a cancer if its target genes are functionally similar to those genes associated with the studied cancer, Li et al. defined a functional consistency score to measure the correlation between microRNA target genes and cancer‐associated genes for the identification of cancer‐related microRNAs.^4^ Moreover, Madden et al. developed an online tool called “CombiROC” to assist finding optimal combination of biomarkers through receiver operating characteristic (ROC) curve analysis.^5^ Recently, machine learning has gradually become more basic and widely used in various fields of research. Zhao et al. used gene expression profiling data and prior knowledge on signaling pathways to identify the dysfunctional pathways in disease conditions; they also performed reverse inference for the identification of cancer‐related microRNAs by microRNA–mRNA regulatory network.^6^ Mukhopadhyay et al. proposed a packaged genetic algorithm for multiobjective optimization and identified microRNAs as potential biomarkers for cancer by support vector machine (SVM) classifiers.^7^ Moreover, combinatorial biomarkers tend to be more efficient and accurate predictors than single biomarkers. Yang et al. developed a method based on the cluster analysis for identifying microRNA biomarkers through the following steps: first differentially expressed microRNAs were detected between studied samples and the control group by statistical *t*‐test. The remaining microRNAs were clustered, and a representative combination of microRNA biomarkers was selected as cancer biomarker using Fisher linear discrimination.^8^ Complex network theory provides a way for researchers to study complex diseases at the systemic level. The network topology can describe the degree of action and contribution of biomolecules to complex systems, such as disease evolution. Based on this theory, Xu et al. built a microRNA target‐dysregulated network and defined four topological features for microRNAs including Dout, NmicroRNA, Rpc‐microRNA, and Rtarpc‐microRNA to measure the regulatory ability of microRNAs in cancers. This model was applied in finding prostate cancer‐associated microRNAs.^9^ Subsequently, Chen et al. constructed a microRNA–microRNA functional similarity network and applied a random walk strategy to predict disease‐associated microRNAs. Instead of using the traditional methods, they adopted the global network similarity measures to optimize candidate microRNAs.^10^

On the other hand, such network‐based methods still face many challenges. The inhibitory effect of microRNAs on its target mRNA translation is mainly based on base‐pairing interaction between the microRNAs and the 3′‐untranslated region (3′‐UTR) of target mRNAs. A microRNA can have many target mRNAs, and a mRNA can also be regulated by multiple microRNAs, leading to a many‐to‐many microRNA–mRNA regulatory mode. Extensive research efforts have focused on the synergistic microRNA regulation and “multiple‐to‐multiple” model between microRNAs and their targets. Meanwhile, the independent regulatory power of individual microRNAs, that is, the “one‐to‐multiple” mode of microRNA‐mRNAs relationship, is less explored. Our study suggested that the predictions of potential microRNA biomarkers with independent regulatory abilities (outlier microRNAs) deserve further investigation.^11^ Based on this theory, the Pipeline of Outlier MicroRNA Analysis (POMA) framework has been developed and applied to the identification of microRNA biomarkers in prostate cancer,^11^ renal clear cell carcinoma,^12^ and acute myeloid leukemia.^13^ However, the POMA framework and other network‐based methods do not conduct in‐depth examination of the functions of target genes, especially the regulatory power of target genes. In this study, we applied our “single‐line mRNA (gene) regulation model” to biomarker microRNA discovery in hepatocellular carcinoma (HCC). The resulting putative biomarker microRNAs were then validated through in‐depth examination of their molecular mechanisms and functions.

## 2 METHODS

### 2.1 Data collection for HCC microRNA/mRNA expressions and interactions

To construct a more comprehensive and reliable human microRNA–mRNA network, we collected expression profiles from the Gene Expression Omnibus (GEO) database, microRNA–mRNA target relationships from multiple microRNA databases, as well as computational prediction of microRNA targets.

HCC microRNA expression data were obtained from the following three datasets in GEO: GSE63046, GSE21279, and GSE36915. GSE63046 contains 24 HCC samples and 24 normal adjacent tissue samples, the latter of which consisted of 15 samples with cirrhosis and nine without cirrhosis.^14^ GSE21279 included 15 different types of liver tissue samples, among which four HCC samples and three normal samples were selected for further analyses.^15^ GSE36915 consists of 68 HCC and 21 nontumor liver tissues.^16^ In this work, we selected nine HCC samples and nine adjacent noncirrhosis tissue samples from GSE63046 dataset and downloaded the preprocessed microRNA expression profiling data for detailed analysis. The other two datasets (GSE21279 and GSE36915) were utilized for the validation study.

In our present study, data extracted from three GEO datasets, namely, GSE14520, GSE25097, and GSE36376, were used for further analyses of the expression correlations between microRNAs and target genes: 225 HCC samples and 220 nontumor liver samples were chosen from GSE14520, which used the Affymetrix HT Human Genome U133A Array platform^17^; 268 HCC tumor and 243 adjacent nontumor samples were selected from GSE25097^18^; the entire collection of 240 HCC samples and 193 adjacent nontumor samples in GSE36376 was included.^19^

For microRNA–mRNA target relationship data, we integrated experimentally validated microRNA–gene interactions from various databases, including miRTarBase,^20^ TarBase,^21^ miRecords,^22^ and miR2Disease.^23^ In addition to curated database of microRNA–target interactions, we downloaded computational microRNA–target prediction data from HOCTAR,^24^ ExprTarget,^25^ and starBase.^26^, ^27^ HOCTAR is a resource that integrates expression profiling data and results predicted by sequence‐based target prediction tools such as PicTar,^28^ TargetScan,^29^ and miRanda.^29^ Moreover, it also uses Gene Ontology to analyze each microRNA‐mediated transcriptional regulation network, thus predicting biological functions of microRNAs. ExprTarget includes microRNA target prediction methods contained in HOCTAR, and also integrates microRNA and mRNA expression datasets of the HapMap cell line.^25^ The starBase provides the information on microRNA–ceRNA, microRNA–ncRNA and protein–RNA interaction networks from large‐scale CLIP‐Seq data.

For gene‐transcription factor (TF) data, we collected data from the article of Vacerizas et al., which included a total of 1843 human TF genes.^30^

### 2.2 Construction of human microRNA–mRNA reference network

For experimentally validated microRNA–mRNA interactions, we mainly selected the microRNA–mRNA interactions verified by low‐throughput experiments (e.g., quantitative polymerase chain reaction [qPCR]). For computational microRNA–target prediction data, we selected the top 50% with highest scores in HOCTAR, the prediction score over 1 in ExprTarget, and the detection credibility readNUM ≥ 10 and BC (Biological complex) ≥ 2 in starBase. Finally, the results of at least two out of the three databases (HOCTAR, ExprTarget, and starBase) must match to positively identify the predicted interaction between microRNA and mRNA. Additionally, we unified all the microRNA names according to the rule in the latest release of the miRBase database (v21).^31^, ^32^

### 2.3 Screening of differentially expressed microRNAs and mRNAs

For the mRNA expression profile data, missing data were imputed by the k‐nearest neighbors imputation approach in R, and the differential expression analysis was applied here, using the method described by Tang et al.^33^ For the microRNA expression profiling data, the Student's *t* test was utilized to calculate the differentially expressed microRNAs between disease groups and control groups, and the *p*‐value threshold for statistical significance was less than 0.05.

### 2.4 Construction of condition‐specific microRNA–mRNA network for HCC

First, the differentially expressed microRNAs and mRNAs were mapped to the human microRNA–mRNA reference network to obtain two HCC‐specific microRNA–mRNA networks, called HCC‐Net1 and HCC‐Net2, respectively. Two indices, namely, novel out degree (NOD) and transcription factor percentage (TFP) values of microRNAs in two condition‐specific microRNA–mRNA interaction networks, were calculated to quantify the power of microRNA regulation and identify candidate microRNA biomarkers for HCC.^11^, ^12^, ^34^, ^35^

NOD is a novel index of the network vulnerability, which represents the number of genes in the network that exclusively targeted by a certain microRNA. NOD embodies the independent regulatory power of individual microRNAs, and these microRNAs are more likely to become the vulnerable components of networks, and reflect changes in disease status more sensitively and accurately. TFP indicates the proportion of TF genes in microRNA‐regulated genes, and provides an important complement to NOD that further extends the identification of microRNA biomarkers model to the biological function level. Because TF is crucial in many biological processes, abnormal expression of TF genes may play important roles in promoting cancer development. When abnormal regulation occurs in a microRNA with a higher TFP value, it may affect the expression of more TF genes and further have an effect on downstream genes leading to changes in the network state eventually. We calculated NOD and TFP values for each microRNA in the HCC‐Net1 and HCC‐Net2, respectively. Those microRNAs with significantly higher NOD and TFP values and *p* value of Wilcoxon signed‐rank test <0.05 were selected as candidate biomarkers for HCC.

### 2.5 Biological function analysis

The biological function analyses (gene ontology or signal pathway enrichment analysis) of candidate microRNAs were performed using DAVID (the Database for Annotation, Visualization and Integrated Discovery) and IPA (Ingenuity pathway analysis) tools. The top 10 most significantly enriched pathways were selected for further literature mining and validation.

### 2.6 Classification performance evaluation

To verify the diagnostic effect of selected microRNAs, we used fivefold cross‐validation cross‐referencing a SVM model in the training dataset to evaluate their diagnostic capabilities. The sensitivity, specificity, and accuracy are used to determine the performance of our method and are calculated as follows:

$$\mathrm{Sensitivity}\;=\;\mathrm{TP}/\left(\mathrm{TP}\;+\;\mathrm{FN}\right),$$

$$\mathrm{Specificity}\;=\;\mathrm{TN}/\left(\mathrm{FP}\;+\;\mathrm{TN}\right),$$

$$\mathrm{Accuracy}\;=\;\left(\mathrm{TN}\;+\;\mathrm{TP}\right)/\left(\mathrm{TP}\;+\;\mathrm{FP}\;+\;\mathrm{TN}\;+\;\mathrm{FN}\right),$$

where TP, TN, FP, and FN represent true positive, true negative, false positive, and false negative, respectively. In addition, ROC curve analysis and area under the curve (AUC) were also applied to measure the classification performance of candidate microRNA biomarkers identified by our method both in the training expression profile dataset and the validation dataset. The SVM model is implemented in an R package “e1071,” and the ROC curve analysis is conducted in an R package “ROCR.”

### 2.7 Cell culture and luciferase‐labeled cells

The HepG2, Huh7, SMMC‐7721, Li‐7, PLC/PRF5, and SK‐Hep‐1 human HCC cell lines; the HL‐7702 (L‐02) normal human liver cell line; and the 293T human embryonic kidney cell line were purchased from the Cell Bank of Type Culture Collection of the Chinese Academy of Sciences (Shanghai, China). The MHCC97L and MHCC97H human HCC cell lines were kindly provided by Dr. Yang Xu (Liver Cancer Institute and Zhongshan Hospital, Fudan University, Shanghai, China). The abovementioned cells were cultured in Roswell Park Memorial Institute (RPMI)‐1640 medium (HyClone, Logan, UT, USA) containing 10% fetal bovine serum (FBS) (Gibco, Gaithersburgh, MD, USA) and 100 U/mL penicillin–streptomycin (Beyotime Biotech, Beijing, China) in an incubator with 5% CO_2_ at 95% humidity and 37°C. For luciferase (Luc) labeling of cells, MHCC97H cells (0.5 × 10^5^ cells/well) were seeded into a 24‐well plate. After 24 h of culture, the cells were infected with lentivirus–Luc–neomycin (Neo) (abm, Richmond, BC, Canada) at a multiplicity of infection of 20 in the presence of enhanced infection solution (GeneChem, Shanghai, China) and 10 μg/mL polybrene (GeneChem). At 72 h after infection, the infected MHCC97H cells were selected using 1000 μg/mL Neo (Beyotime Biotech) to obtain Luc‐labeled MHCC97H cells.

### 2.8 Reverse transcription‐qPCR and in situ hybridization analyses of microRNAs

The total RNAs derived from 28 pairs of snap‐frozen human HCC tumor tissues and adjacent nontumor (normal) liver tissues (Shanghai Eastern Hepatobiliary Surgery Hospital, Shanghai, China) as well as human HCC cells (HepG2, HuH7, SMMC‐7721, Li‐7, PLC/PRF5, SK‐Hep‐1, MHCC97L, and MHCC97H) and human liver cells (L‐02) were purified using a miRNeasy mini kit (Qiagen, Hilden, Nordrhein‐Westfalen, Germany), and then reversely transcribed to first‐strand complementary DNAs (cDNAs) with a miScript II RT kit (Qiagen). The qPCR was performed using a miScript SYBR Green PCR kit (Qiagen) in a LightCycler 96 Instrument (Roche, Penzberg, Upper Bavaria, Germany). Primers used for analysis of microRNAs (miR‐25‐3p, miR‐101‐3p, miR‐221‐3p, miR‐378a‐3p, miR‐381‐3p, and miR‐490‐3p) were synthesized by Sangon Biotech (Shanghai, China). Primer sequences were shown in File S1. The reaction mix of qPCR contained the following components: 1 μL 10× miScript Universal Primer, 1 μL 10 μM each microRNA‐specific primer or internal reference primer U6, 5 μL SYBR Green, 2 μL RNase‐free water, and 1 μL cDNA template. The qPCR condition was an initial denaturation step (95°C for 15 min) followed by amplification and quantification steps repeated for 40 cycles (95°C for 15 s, 55°C for 30 s, and 70°C for 30 s). The relative expression of microRNAs was normalized to U6 and calculated by a Δ*CT* ^36^ or 2^−ΔΔ^ *^CT^* ^37^ method as reported previously.

The expression of miR‐378a‐3p in human HCC tumor tissues was further analyzed by in situ hybridization (ISH) using a human HCC tissue microarray (TMA) section (cat. no. HLivH180Su06; 90 cases, 90 paired human HCC tumor and adjacent normal liver tissues, 180 dots) (OUTDO Biotech, Shanghai, China). The ISH was performed with 5′‐digoxigenin‐ and 3′‐digoxigenin‐labeled hsa‐miR‐378a‐3p miRCURY locked nucleic acid detection probe (Exiqon, Vedbaek, Denmark) following the manufacturer's instructions. After hybridization, the section was incubated with mouse anti‐digoxigenin (1:200) (cat. no. ab420; abcam, Cambridge, MA, USA) primary antibody followed by alkaline phosphatase‐conjugated goat anti‐mouse IgG (1:5000) (cat. no. ab205719; abcam) secondary antibody. The signal of hybridization was then detected by 5‐bromo‐4‐chloro‐3‐indolyl phosphate/nitro‐blue‐tetrazolium. Finally, the slides were counterstained with nuclear fast red and coverslip mounted. The percentage of positive cells (≤5%, 0; 5%–25%, 1; 25%–50%, 2; 50%–75%, 3; and ≥75%, 4) and the intensity (negative, 0; weak, 1; moderate, 2; and strong, 3) were used for ISH scoring. The scores were then added to produce a weighted ISH scoring (0–1, –; 2–3, +; 4–5, ++; and 6–7, +++) for each specimen. It was considered as high expression in tissue specimen when the final weighted score was ≥4 (++ and +++).

### 2.9 Cell proliferation assays

Cell proliferation was evaluated through cell counting kit‐8 (CCK‐8) and colony formation assays in vitro. Briefly, for a CCK‐8 assay, MHCC97H or SMMC‐7721 cells (1 × 10^4^ cells/well) were seeded into 96‐well plates. Twenty‐four hours later, the MHCC97H cells were treated with agomiR‐378a‐3p or agomiRcontrol (RiboBio, Guangzhou, Guangdong, China), whereas the SMMC‐7721 cells were treated with antagomiR‐378a‐3p or antagomiRcontrol (RiboBio), at a final concentration of 200 nM following the company's suggestions. Cell viability of MHCC97H‐agomiR‐378a‐3p versus MHCC97H‐agomiRcontrol (used as a control) as well as SMMC‐7721‐antagomiR‐378a‐3p versus SMMC‐7721‐antagomiRcontrol (used as a control) was then evaluated with CCK‐8 (Beyotime Biotech) on day 1, 2, 3, and 4 after treatment and read at 450 nm in an automatic microplate reader (Thermo Fisher Scientific, Waltham, MA, USA) according to the manufacturer's guidelines. For a colony formation assay, the generated MHCC97H‐agomiR‐378a‐3p versus MHCC97H‐agomiRcontrol and SMMC‐7721‐antagomiR‐378a‐3p versus SMMC‐7721‐antagomiRcontrol cells (400 cells/well) were reseeded into six‐well plates. After 2–3 weeks of culture, the cells were stained with 0.5% crystal violet for 10–30 min after fixation with 4% paraformaldehyde for 15 min at room temperature. The clonogenic ability of these cells was then analyzed.

### 2.10 Microsphere‐forming assay

After treatment of MHCC97H or SMMC‐7721 cells with agomiR‐378a‐3p versus agomiRcontrol or antagomiR‐378a‐3p versus antagomiRcontrol (200 nM) for 48 h, the generated MHCC97H‐agomiR‐378a‐3p versus MHCC97H‐agomiRcontrol as well as SMMC‐7721‐antagomiR‐378a‐3p versus SMMC‐7721‐antagomiRcontrol cells were redispensed into six‐well ultralow adherent plates (Corning, Corning, NY, USA) at a density of 4 × 10^3^ cells/2 mL complete MammoCult™ medium (MammoCult™ basal medium plus 10% MammoCult™ proliferation supplement) (STEMCELL Technologies, Vancouver, BC, Canada) per well. One week after incubation, the number of tumor microspheres was counted.

### 2.11 Scratch assay

The MHCC97H or SMMC‐7721 cells (5 × 10^5^ cells/well) were seeded into six‐well plates, incubated overnight, and then treated with agomiR‐378a‐3p versus agomiRcontrol or antagomiR‐378a‐3p versus antagomiRcontrol (200 nM). After 48 h of treatment, scratches were generated across the entire diameter of wells. Progression of tumor cell migration was observed and photographed at a low‐power field (×100) under microscopy at 0 and 24 h after scratches. The ImageJ software (National Institutes of Health, Bethesda, MA, USA) was then used to calculate the migration distance to analyze the migration ability of these cells.

### 2.12 Transwell invasion assay

The MHCC97H‐agomiR‐378a‐3p versus MHCC97H‐agomiRcontrol and SMMC‐7721‐antagomiR‐378a‐3p versus SMMC‐7721‐antagomiRcontrol cells were resuspended in serum‐free medium supplemented with 0.5% bovine serum albumin at a density of 1 × 10^6^ cells/mL. The cell suspensions (1 × 10^5^ cells/100 μL) were seeded onto the upper chamber of Matrigel (Corning)‐coated 24‐well Transwell filters (8 μm pore size) (Merck Millipore, Billerica, MA, USA). The lower chamber was filled with 500 μL of medium containing 10% FBS. After 24 h of incubation, tumor cells invading into the bottom side of insert were fixed with 4% paraformaldehyde, stained with 0.5% crystal violet, and counted in five randomly selected high‐power fields (×200) under microscopy. The invasive ability of these cells was then analyzed.

### 2.13 Tumor xenograft experiments

Four‐week‐old female athymic BALB/c nude mice were purchased from Shanghai SLAC Laboratory Animal Co., Ltd. (Shanghai, China). The mice were maintained in the animal facility at Soochow University (Suzhou, China). All animal experiments in the study were approved by the Animal Research Ethics Committee of Soochow University. For a subcutaneous (s.c.) tumor‐forming experiment, the Luc‐labeled MHCC97H‐agomiR‐378a‐3p versus MHCC97H‐agomiRcontrol cells (2 × 10^6^ cells/100 μL phosphate‐buffered saline [PBS] per mouse; six mice per group) were subcutaneously (s.c.) injected into nude mice. When the tumor grew to 0.5 cm in diameter, it was considered to be tumor formation and the time was recorded. The tumor growth was further monitored by measuring tumor volume and detecting luciferin signal in a Caliper IVIS Lumina II system (Caliper Life Sciences, Hopkinton, MA, USA) every week until 4 weeks after inoculation. The tumor volume was calculated by a formula: *V* = *A* × *B* ^2^/2, where *A* is the long diameter and *B* is the short diameter. Four weeks after tumor cell inoculation, the mice were killed and the tumors were removed from the body and weighted. For a s.c. tumor treatment experiment, the Luc‐labeled MHCC97H cells (2 × 10^6^ cells/100 μL PBS per mouse; 12 mice) were s.c. injected into nude mice, and then subjected to treatment with agomiR‐378a‐3p or agomiRcontrol (six mice per group) via intratumoral injection (2 nmol per time) when the tumors grew to around 0.5 cm in diameter. The treatment was performed once every 3 days for four times according to the company's protocols. For an orthotopic tumor treatment assay, the Luc‐labeled MHCC97H cells (2 × 10^6^ cells/100 μL PBS) were s.c. injected into the nude mice. When the tumor grew to a diameter of l cm, the s.c. tumor was resected, soaked in PBS, cut into 1–2 mm^3^ tumor size, and implanted orthotopically in the right lobes of the livers of the nude mice. One week after the orthotopic transplantation, the growth of orthotopic tumors was examined by a Caliper IVIS Lumina II system to detect luciferin signal. Those mice that carried orthotopic tumors were then assigned to two groups (six mice per group). Each group was treated with 5 nmol of agomiR‐378a‐3p or agomiRcontrol each time by tail vein injection. The treatment was also performed once every 3 days for four times. The growth of s.c. and orthotopic tumors was tracked as described above before (week 0) and every week (week 1–4) after treatment. In addition, the lung tissues and the s.c./orthotopic tumor tissues from these tumors‐bearing mice were fixed in 10% neutral formalin and embedded in paraffin. The lung and tumor tissue sections (3‐μm thick) were then prepared and used for hematoxylin and eosin (H&E) analysis of lung metastatic nodules and immunohistochemistry (IHC) analysis of β‐catenin, respectively. For a tumor lung metastasis assay, the Luc‐labeled MHCC97H‐agomiR‐378a‐3p versus MHCC97H‐agomiRcontrol cells (2 × 10^6^ cells/200 μL PBS per mouse; six mice per group) were injected into nude mice through tail vein. Four weeks later, the mice were killed to harvest their lung tissues for H&E analysis of lung metastatic nodules.

### 2.14 Identification and validation of miR‐378a‐3p target gene

Seven genes including PLAG1, SULF1, SLC7A6, NPAT, HDAC4, PLAGL2, and GLI3 were identified as potential targets of miR‐378a‐3p from the microRNAorg, PITA, and TargetScan databases. The 3′UTR dual Luc reporter plasmids for PLAG1, SULF1, SLC7A6, NPAT, HDAC4, PLAGL2, and GLI3 (termed PLAG1‐3′UTR, SULF1‐3′UTR, SLC7A6‐3′UTR, NPAT‐3′UTR, HDAC4‐3′UTR, PLAGL2‐3′UTR, and GLI3‐3′UTR) were constructed by GeneChem. The 293T cell line was used as a target cell for detection of Luc activity. In 24‐well plates, the 293T cell was cotransfected with miR‐378a‐3p mimics or miRNA mimics negative control (NC) (used as a control) (200 nM) and PLAG1‐3′UTR, SULF1‐3′UTR, SLC7A6‐3′UTR, NPAT‐3′UTR, HDAC4‐3′UTR, PLAGL2‐3′UTR, or GLI3‐3′UTR (1 μg per well) by Lipofectamine 2000 (Thermo Fisher Scientific). After 48 h of transfection, the effect of miR‐378a‐3p on candidate target genes was preliminarily analyzed by measuring the Luc activity with a Luc Assay System (Promega, Madison, WI, USA) based on company's protocols. The PLAGL2‐3′UTR mutant dual Luc reporter plasmid was further constructed (GeneChem) because miR‐378a‐3p had the most potent inhibitory effect on PLAGL2 gene. To validate the targeting effect of miR‐378a‐3p on PLAGL2 as well as the targeting site, different concentrations (200, 100, 50, and 25 nM) of miR‐378a‐3p mimics or miRNA mimics NC and 1 μg of PLAGL2‐3′UTR or PLAGL2‐3′UTR mutant were used for cotransfection into 293T cells to analyze the Luc activity.

To further confirm the targeting effect of miR‐378a‐3p on PLAGL2 in HCC cells, the MHCC97H‐agomiR‐378a‐3p versus MHCC97H‐agomiRcontrol and SMMC‐7721‐antagomiR‐378a‐3p versus SMMC‐7721‐antagomiRcontrol cells were generated for reverse transcription (RT)‐qPCR and western blot analyses of PLAGL2. Briefly, the total RNAs and lysates of these cells were purified with a MiniBEST universal RNA extraction kit (TaKaRa, Dalian, Liaoning, China) and mammalian cell lysis buffer with phenylmethylsulfonyl fluoride (1 mM) (Beyotime Biotech), respectively. The RNAs were reversely transcribed to cDNAs with a 5× All‐In‐One RT MasterMix kit (abm), and then subjected to qPCR analysis of PLAGL2 with the EvaGreen 2× qPCR MasterMix kit (abm) using human PLAGL2‐specific primer pair (β‐actin used as an internal reference) (Sangon Biotech) (File S1). The qPCR was conducted at 95°C for 10 min followed by 40 cycles at 95°C for 15 s and 60°C for 30 s. The relative level of PLAGL2 mRNA was standardized to β‐actin and calculated by a 2^−ΔΔ^ *^CT^*.^37^ After determination of protein concentration using a bicinchoninic acid protein assay kit (Beyotime Biotech), the lysates were loaded (50 μg per lane) and separated by a 12% sodium dodecyl sulfate–polyacrylamide gel electrophoresis (Beyotime Biotech), and then transferred onto polyvinylidene difluoride membranes (Merck Millipore). The membranes were blocked with 5% fat‐free milk, and then subjected to immunoblotting analysis for PLAGL2 or β‐actin (used as a loading control) by incubation with rabbit anti‐PLAGL2 (1:1000) (cat. no. GTX32095; GeneTex, Irvine, CA, USA) or anti‐β‐actin (1:3000) (cat. no. YM3214; ImmunoWay, Plano, TX, USA) primary antibody at 4°C overnight. Following incubation with horseradish peroxidase (HRP)‐conjugated goat anti‐rabbit IgG (1:12000) (cat. no. RS0002; ImmunoWay) secondary antibody at room temperature for 1 h, the signal was developed with a BeyoECL Star kit (Beyotime Biotech), scanned by Gel Imaging System (P&Q Science & Technology, Shanghai, China), and quantified by ImageJ software.

### 2.15 Analyses of β‐catenin in vitro

The nuclear lysates derived from MHCC97H‐agomiR‐378a‐3p versus MHCC97H‐agomiRcontrol and SMMC‐7721‐antagomiR‐378a‐3p versus SMMC‐7721‐antagomiRcontrol cells were prepared using a nuclear and cytoplasmic protein extraction kit (Beyotime Biotech), and then subjected to western blot analysis for β‐catenin or Histone H3 (loading control) using rabbit anti‐β‐catenin (1:2000) (cat. no. 8480; Cell Signaling Technology, Danvers, MA, USA) or anti‐Histone H3 (1:2000) (cat. no. 9717; Cell Signaling Technology) primary antibody, as described above. The total lysates of these cells were also prepared and subjected to western blot analysis for β‐catenin or β‐actin (loading control).

The MHCC97H or SMMC‐7721 cells (2 × 10^5^ cells/well) were seeded on coverslips in six‐well plates overnight, and then treated with agomiR‐378a‐3p versus agomiRcontrol or antagomiR‐378a‐3p versus antagomiRcontrol (200 nM). After treatment for 48 h, the cells were subjected to immunofluorescence analysis of β‐catenin. Briefly, the coverslips were fixed in 4% paraformaldehyde, permeabilized with PBS containing 0.2% Triton X‐100 and blocked by 5% normal goat serum in PBS. The coverslips were then incubated with rabbit anti‐β‐catenin (1:200) primary antibody followed by incubation with Alexa Fluor 647‐conjugated goat anti‐rabbit IgG (1:500) (cat. no. ab150079; abcam) secondary antibody. Finally, the coverslips were prevented from fade using antifade mounting medium with 4′,6‐diamidino‐2‐phenylindole (DAPI) (Beyotime Biotech) and put onto slides. The immunofluorescence was then observed under confocal microscopy (Leica, Wetzlar, Hesse‐Darmstadt, Germany).

The MHCC97H or SMMC‐7721 cells (0.5 × 10^5^ cells/well) were seeded into a 24‐well plate and cultured overnight. The cells were transfected with 1 μg of TCF/LEF1‐Luc report plasmid (Genomeditech, Shanghai, China) per well by Lipofectamine 2000, and then selected with Neo (1000 μg/mL) to generate MHCC97H‐TCF/LEF1‐Luc and SMMC‐7721‐TCF/LEF1‐Luc transgenic cells for detection of β‐catenin's transcriptional activity by Luc reporter assay. Subsequently, the MHCC97H‐TCF/LEF1‐Luc or SMMC‐7721‐TCF/LEF1‐Luc cells (0.5 × 10^5^ cells/well) were seeded into 24‐well plates. After 24 h of culture, the MHCC97H‐TCF/LEF1‐Luc cells were treated with agomiR‐378a‐3p versus agomiRcontrol (200 nM), whereas the SMMC‐7721‐TCF/LEF1‐Luc cells were treated with antagomiR‐378a‐3p versus antagomiRcontrol (200 nM). After 48 h of treatment, the Luc activity of these cells was analyzed by a Luc Assay System.

### 2.16 Small interfering RNA knockdown assays

The SMMC‐7721 cells were treated with either 200 nM of antagomiR‐378a‐3p or antagomiRcontrol. The antagomiR‐378a‐3p‐treated SMMC‐7721 cells were also simultaneously transfected with human siTCF4 (TCF4 small interfering RNA [siRNA]), human LEF1 siRNA (siLEF1), human PLAGL2 siRNA (siPLAGL2), or control siRNA (sicontrol) (RiboBio) at a final concentration of 100 nM by HiPerFect transfection reagent (Qiagen). The siTCF4‐ and sicontrol‐transfected SMMC‐7721‐antagomiR‐378a‐3p cells were subjected to western blot analysis for TCF4 (1:2000) (cat. no. 2566; Cell Signaling Technology) and β‐actin (loading control), whereas the siLEF1‐ and sicontrol‐transfected SMMC‐7721‐antagomiR‐378a‐3p cells were subjected to western blot analysis for LEF1 (1:2000) (cat. no. GTX129186; GeneTex) and β‐actin (loading control). The siTCF4‐, siLEF1‐ and sicontrol‐transfected SMMC‐7721‐antagomiR‐378a‐3p cells as well as the siRNA‐untransfected SMMC‐7721‐antagomiR‐378a‐3p and SMMC‐7721‐antagomiRcontrol cells were then subjected to CCK‐8, scratch, and Transwell invasion assays. In addition, the siPLAGL2‐ and sicontrol‐transfected SMMC‐7721‐antagomiR‐378a‐3p cells as well as the siRNA‐untransfected SMMC‐7721‐antagomiR‐378a‐3p and SMMC‐7721‐antagomiRcontrol cells were subjected to western blot analysis for PLAGL2, β‐catenin and β‐actin/Histone H3 (total/nuclear loading control), and Luc reporter assay of β‐catenin‐mediated transcriptional activity as described above.

### 2.17 IHC analysis

The expression of PLAGL2 and β‐catenin in human HCC tumor tissues was determined by IHC analysis using human HCC TMA sections. The sections were deparaffinized and rehydrated. Antigen retrieval was performed by microwaving the slides in 0.01 M citrate buffer (pH 6.0) for 10 min. Endogenous peroxidase activity was quenched by treatment with 3% H_2_O_2_ for 30 min followed by incubation with normal goat serum for 15 min. Subsequently, the sections were incubated with rabbit anti‐PLAGL2 (1:500) and anti‐β‐catenin (1:100) primary antibody in a humidity chamber overnight at 4°C. The sections were then incubated with HRP‐conjugated goat anti‐rabbit IgG (1:200) (cat. no. G1213; Servicebio, Wuhan, Hubei, China) secondary antibody for 1 h at room temperature and immunostaining signal was detected by 3′,3‐diaminobenzidine. Finally, the slides were counterstained with H&E and coverslip mounted. The expression level of PLAGL2 and β‐catenin was evaluated by a weighted IHC score similarly as described above in the ISH analysis. In addition, the HCC transplanted tumor tissue sections were subjected to IHC analysis of β‐catenin.

### 2.18 Statistical analyses for experimental results

The statistical analyses such as Student's *t* test, analysis of variance, Mann–Whitney *U* test, Pearson's *χ* ^2^ test, and Log‐rank test were performed with SPSS13.0 (SPSS, Chicago, IL, USA). A *p*‐value of less than 0.05 (*p < 0.05 and **p < 0.01) was considered to be statistically significant.

## 3 RESULTS

### 3.1 Construction of human microRNA–mRNA reference network

**FIGURE 1:**
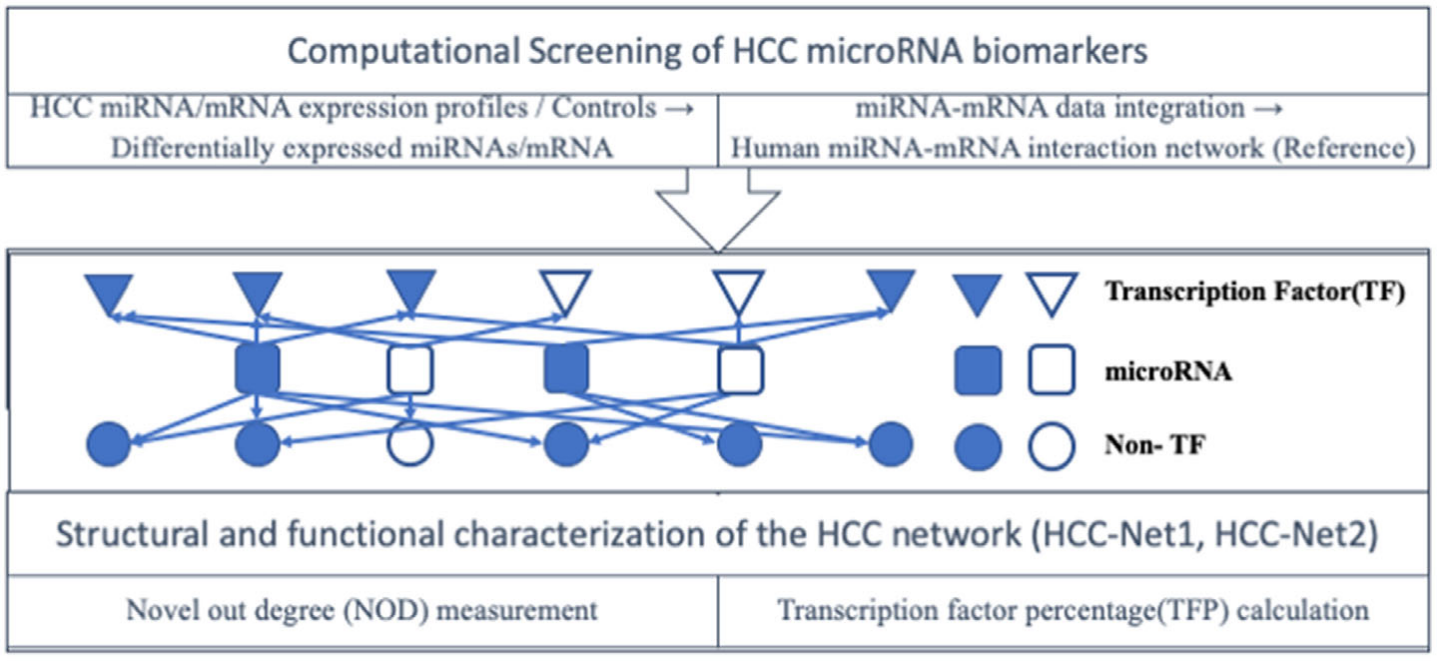
The flowchart of the computational screening of the putative microRNA biomarkers for the HCC diagnosis

The flowchart for the computational screening of HCC microRNA biomarker is shown in Figure 1. In this study, we integrated experimentally validated microRNA–gene interactions from four databases (miRTarBase, TarBase, miRecords, and miR2Disease), and only selected those microRNAs with consistent results in at least two out of the three computational microRNA–target prediction methods (HOCTAR, ExprTarget, and starBase). In total, we obtained a human microRNA–mRNA reference network containing 618 microRNAs, 9526 target genes, and 48,868 microRNA–mRNA target relationships. In this directional binary network, the average degree of microRNAs was 79, and the average degree of target genes was 5 in the microRNA–mRNA network. Top 10 most significantly enriched pathways were selected for further literature mining and validation.

Moreover, the degree of microRNAs also matches the power law distribution, which means that compared to the remaining vast majorities, only a small number of microRNAs regulate more target genes in the networks. The NOD and TFP value distribution features of microRNAs show about 66.3% (410/618) of microRNAs with a NOD value of greater than 0, meaning these microRNAs are more likely to have greater independent regulatory power. Furthermore, previously reported microRNA biomarkers generally have significantly higher NOD and TFP values than the remaining microRNAs.^1^, ^11^, ^35^

### 3.2 Screening of differentially expressed microRNAs and mRNAs

To more accurately identify differentially expressed mRNAs, six methods (*t*‐test, COPA, OS, ORT, MOST, and LSOSS) were used to screen differentially expressed genes (DEGs) in three HCC datasets (GSE14520, GSE25097, and GSE36376). The percentage of overlapped DEGs obtained by each method in each dataset was calculated to select the best method. Considering the robustness of results in different datasets, the top 40% of the DEGs identified by each method were selected for comparison in this study. Therefore, 3341 DEGs screened by LSOSS were selected for detailed analyses. *t*‐test was performed to select differentially expressed microRNAs in HCC between nine HCC samples and nine normal adjacent tissue samples with cirrhosis (GSE63046), resulting in 149 differentially expressed microRNAs in total (*p* < 0.05). Among them, 89 exhibit higher expression and 60 show lower expression in HCC.

### 3.3 Construction of condition‐specific microRNA–mRNA network in HCC

To construct condition‐specific microRNA–mRNA network in HCC, differentially expressed microRNAs and mRNAs were mapped to the human microRNA–mRNA reference network, producing two HCC networks (HCC‐Net1 and HCC‐Net2). HCC‐Net1 contains 85 microRNAs, 4852 target genes, and 9428 microRNA–mRNA target relationships. The average degree of microRNAs is 111, and the average degree of mRNAs is equal to 2. Similarly, HCC‐Net2 includes 520 microRNAs, 2697 genes, and 17,889 microRNA–mRNA target relationships. The average degrees of microRNAs and genes are 34 and 7. HCC‐Net2 has less number and lower average degree of microRNAs than HCC‐Net1, possibly because the expression of target genes in HCC is not considered in the screening process of HCC‐Net1, leading to more target genes in the microRNA–mRNA reference network being included. Moreover, an overlapping analysis showed that 76 out of 85 microRNAs in HCC‐Net1 were overlapped in HCC‐Net2. This demonstrated consistency between microRNAs identified in HCC‐Net1 and HCC‐Net2, thus guaranteeing the rationality of subsequent analyses.

### 3.4 Screened HCC microRNA biomarkers

In HCC‐Net1, compared to the controls, 33 microRNAs show significantly higher NOD values (*p *< 0.05), and 11 exhibit significantly higher TFP values (*p *< 0.05), as listed in File S2. By the same criteria, 211 microRNAs with significantly higher NOD values (*p *< 0.05) and 95 with significantly higher TFP values (*p *< 0.05) were found in HCC‐Net2 (File S3). Finally, six overlapping microRNAs between HCC‐Net1 and HCC‐Net2 were identified as potential HCC microRNA biomarkers. Among them, miR‐25‐3p and miR‐221‐3p were overexpressed in HCC, and miR‐101‐3p, miR‐378a‐3p, miR‐490‐3p, and miR‐381‐3p were underexpressed in comparison with genes of normal liver tissue, as shown in Table 1.

**TABLE 1:** HCC microRNA biomarkers predicted by our computational screening

microRNA	NOD1	TFP1	NOD2	TFP2	Expression	Npm	Function
miR‐25‐3p	149	0.1703	1	0.1366	Up	2	Cell proliferation Cell wall adhesion
miR‐221‐3p	33	0.1481	1	0.1556	Up	3	Cell apoptosis and proliferation Tumor multifocality and infiltration
miR‐101‐3p	57	0.1642	2	0.2162	Down	7	Tumor suppression Apoptosis, proliferation, and migration
miR‐378a‐3p	57	0.1477	1	0.119	Down	0	Cell proliferation and migration
miR‐490‐3p	48	0.1449	1	0.1607	Down	0	Cell proliferation, migration and infiltration, epithelial–mesenchymal transition
miR‐381‐3p	37	0.1765	1	0.1143	Down	0	N/A

Abbreviations: N/A, not available; NOD1/NOD2 and TFP1/TFP2, NOD and TFP measured in HCC‐Net1 and HCC‐Net2, respectively; Npm, number of PubMed citations as potential biomarkers of HCC.

### 3.5 Biological function analysis of predicted HCC microRNA biomarker

**FIGURE 2:**
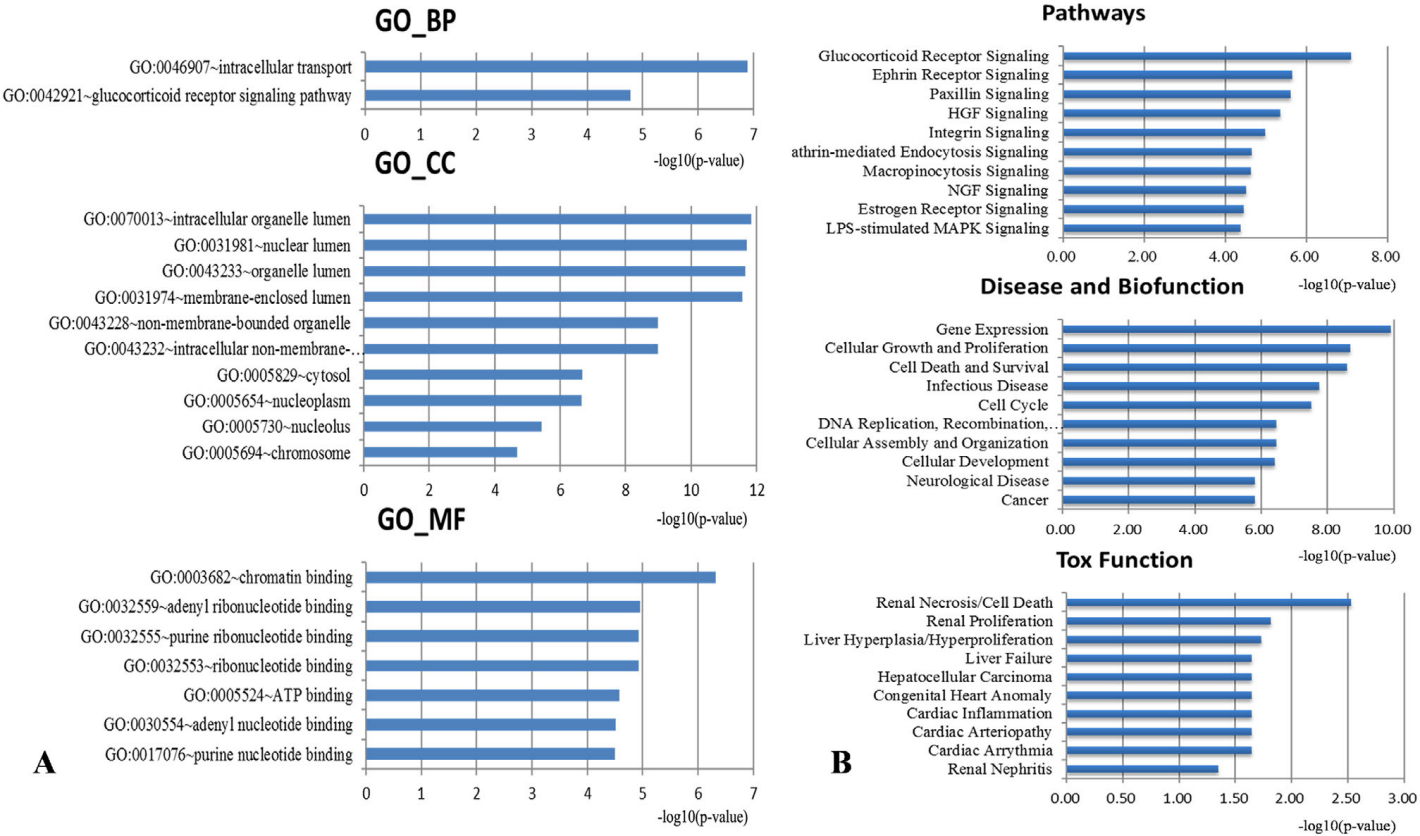
Functional analysis of screened microRNA biomarkers. (A) Ontology enrichment analysis of target genes of the screened microRNA. Here GO_BP, GO_CC, and GO_MF represent biological process, cell composition, and molecular function, respectively. (B) IPA enrichment analysis of target genes of the microRNA biomarkers

To investigate the biological properties of six candidate microRNA biomarkers, we carried out an enrichment analysis for a total of 433 target genes using DAVID and IPA. As shown in Figure 2A, there are two GO terms at biological process level, 10 GO terms at cellular component level, and seven GO terms at molecular function level that are statistically enriched for target genes (*p *< 0.05). Among them, glucocorticoid receptor (GR) is an important signal integrator in liver metabolism and physiological stress. In mice, impairment of GR signaling causes steatosis and HCC.^38^ The significantly enriched GO categories include cellular components from various types of lumens and organelles in both cytoplasm and nucleus. Molecular binding is the most significantly enriched molecular function, which includes chromatin binding, adenyl ribonucleotide binding, ribonucleotide binding, purine ribonucleotide binding, ATP binding, adenyl nucleotide binding, and purine nucleotide binding.

Moreover, Figure 2B listed the top 10 significantly enriched terms in signal transduction pathways, disease and biological functions, and toxic functions using IPA (*p *< 0.05). Notably, the GR signaling pathway ranked first among all 115 significant enriched signal transduction pathways. Other top 10 enriched signal transduction pathways include Ephrin receptor signaling, Paxillin signaling, Hepatocyte growth factor signaling, Integrin signaling, and two types of pinocytosis signals (clathrin‐mediated pinocytosis and macrophage), NGF signaling, estrogen receptor signaling, and LPS‐stimulated MAPK signaling pathway. Importantly, most signal pathways we identified were closely related to HCC. For instance, the impairment of clathrin‐mediated endocytosis via cytoskeletal change by epithelial to fibroblastoid conversion is associated with the des‐gamma‐carboxy prothrombin production in HCC^39^; Hepatocyte growth factor stimulates the formation and migration of HCC cells^40^, ^41^; Estrogen receptor signaling plays an important role in the induction of HCC^42^; LPS‐TLR4 signaling promotes cancer cell survival and proliferation by regulating the activity of the MAPK signaling pathway.^43^ Moreover, most of the significantly enriched entries in disease and biofunction are associated with hallmarks of cancer, including cellular growth and proliferation, cell death and survival, cell cycle, and DNA replication rearrangement, and repair (i.e., DNA replication and recombination).^44^ And as shown in Figure 2B, liver hyperplasia/hyperproliferation, liver failure, and HCC are the top five significantly enriched entries in toxic function.

In summary, the functional enrichment analysis revealed crucial roles of our predicted microRNA targets in the development of HCC, suggesting these microRNAs might serve as good HCC biomarkers.

### 3.6 Classification evaluation of candidate microRNA biomarkers in HCC

**FIGURE 3:**
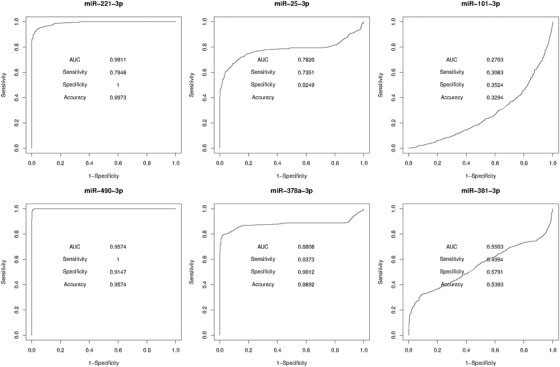
ROC curves of the screened microRNA biomarkers tested with the GSE63046 dataset

**FIGURE 4:**
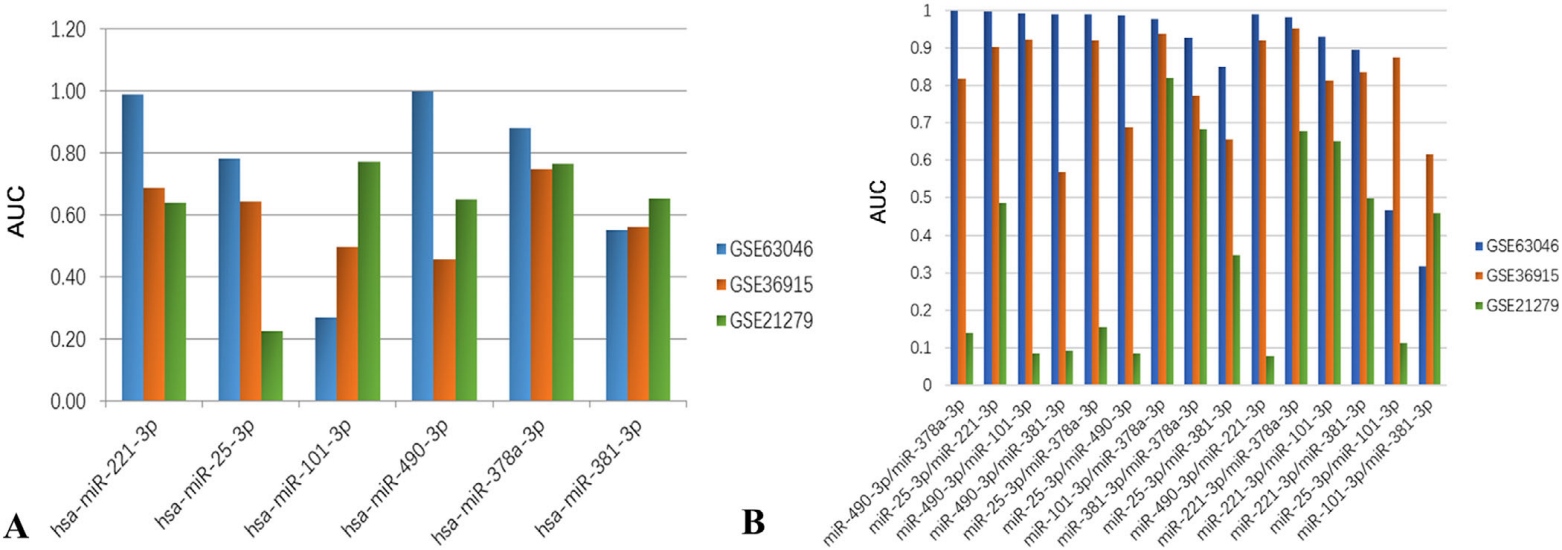
Prediction performance characterized with area under the curve (AUC) values of the screened microRNA biomarkers tested with the three data sets. (A) The performance of single microRNA biomarkers. (B) The performance of the combinatorial microRNA biomarkers

To evaluate the diagnostic values of these six microRNAs, we performed ROC curve analysis and calculated the AUC value in GSE63046, GSE21279, and GSE36915. For the data in GSE63046, Figure 3 showed that miR‐221‐3p, miR‐490‐3p, miR‐378a‐3p, and miR‐25‐3p all had a discriminating power of AUC values larger than 0.7 between HCC patient samples and nontumor samples. For the data in GSE21279 and GSE36915, as shown in Figure 4A, the AUC values of miR‐221‐3p and miR‐378a‐3p in both datasets were greater than 0.6, whereas the AUC value of miR‐490‐3p was only greater than 0.6 in GSE21279. Interestingly, the AUC values of miR‐101‐3p and miR‐381‐3p in GSE63046 were both less than 0.6 and were greater than 0.6 in GSE21279. In addition, the AUC value of miR‐25‐3p was only greater than 0.6 in GSE36915. Therefore, we concluded that miR‐221‐3p and miR‐378a‐3p have a better robustness and classification consistency for universal diagnosis for HCC. The other four microRNAs showed the discriminating power on different datasets, implying their strong classification ability as a personalized medicine model for some specific samples due to the heterogeneity of cancer.

To eliminate the interfering effect, we further assessed various combinations of multiple diagnostic microRNA biomarkers for their diagnostic values. The result of two microRNA combinations showed that about 86.7% (13/15) of microRNA combinations have an AUC greater than 0.8, among which the AUC values of top nine microRNA combinations were greater than those of single microRNA biomarkers (Table 2). Notably, the AUC values of eight microRNA combinations, including miR‐25‐3p/miR‐221‐3p, miR‐490‐3p/miR‐101‐3p, miR‐490‐3p/miR‐381‐3p, miR‐25‐3p/miR‐378a‐3p, miR‐25‐3p/miR‐490‐3p, miR‐101‐3p/miR‐378a‐3p, miR‐381‐3p/miR‐378a‐3p, and miR‐25‐3p/miR‐381‐3p, increased by 10% compared to the average AUC values of single microRNAs themselves. In the same manner, we conducted ROC analysis for above 15 combination microRNAs biomarkers in GSE21279 and GSE36915 datasets. We found that the AUC values of four microRNA combinations, including miR‐101‐3p/miR‐378a‐3p, miR‐381‐3p/miR‐378a‐3p, miR‐221‐3p/miR‐378a‐3p, and miR‐221‐3p/miR‐101‐3p, in both two datasets were greater than 0.6 (Figure 4B). Particularly, the AUC value of miR‐101‐3p/miR‐378a‐3p and miR‐381‐3p/miR‐378a‐3p combination not only had an increase of 20% relative to the average AUC values of single microRNAs themselves in GSE63046, but also showed the increase in two validation datasets. In summary, miR‐101‐3p/miR‐378a‐3p and miR‐381‐3p/miR‐378a‐3p exhibit better diagnostic performance for HCC prediction.

**TABLE 2:** Prediction performance of combined microRNA biomarkers

microRNA	Sensitivity	Specificity	Accuracy	AUC	Mean AUC
miR‐490‐3p/miR‐378a‐3p	0.9540	1.0000	0.9770	0.9993	0.9189
miR‐25‐3p/miR‐221‐3p	0.8406	1.0000	0.9203	0.9985	0.8866
miR‐490‐3p/miR‐101‐3p	0.9742	0.8967	0.9355	0.9917	0.6139
miR‐490‐3p/miR‐381‐3p	0.9596	0.9001	0.9299	0.9909	0.7539
miR‐25‐3p/miR‐378a‐3p	0.9091	0.9641	0.9366	0.9888	0.8314
miR‐25‐3p/miR‐490‐3p	0.9832	0.8653	0.9242	0.9885	0.8697
miR‐101‐3p/miR‐378a‐3p	0.8777	0.9843	0.9310	0.9784	0.5756
miR‐381‐3p/miR‐378a‐3p	0.8406	0.9158	0.8782	0.9264	0.7156
miR‐25‐3p/miR‐381‐3p	0.7733	0.8418	0.8075	0.8495	0.6662
miR‐490‐3p/miR‐221‐3p	0.9574	0.8844	0.9209	0.9889	0.9743
miR‐221‐3p/miR‐378a‐3p	0.9080	0.9787	0.9433	0.9822	0.9360
miR‐221‐3p/miR‐101‐3p	0.8171	0.8833	0.8502	0.9289	0.6307
miR‐221‐3p/miR‐381‐3p	0.8608	0.8732	0.8670	0.8960	0.7707
miR‐25‐3p/miR‐101‐3p	0.4568	0.4579	0.4574	0.4671	0.5262
miR‐101‐3p/miR‐381‐3p	0.3558	0.3715	0.3636	0.3157	0.4103

*Note*: The combination of microRNAs with underscores indicates that their AUC is larger than that of a single microRNA. The mean AUC value represents the average of AUC values of the two microRNAs.

### 3.7 Verification of bioinformatics prediction of microRNAs in HCC

**FIGURE 5:**
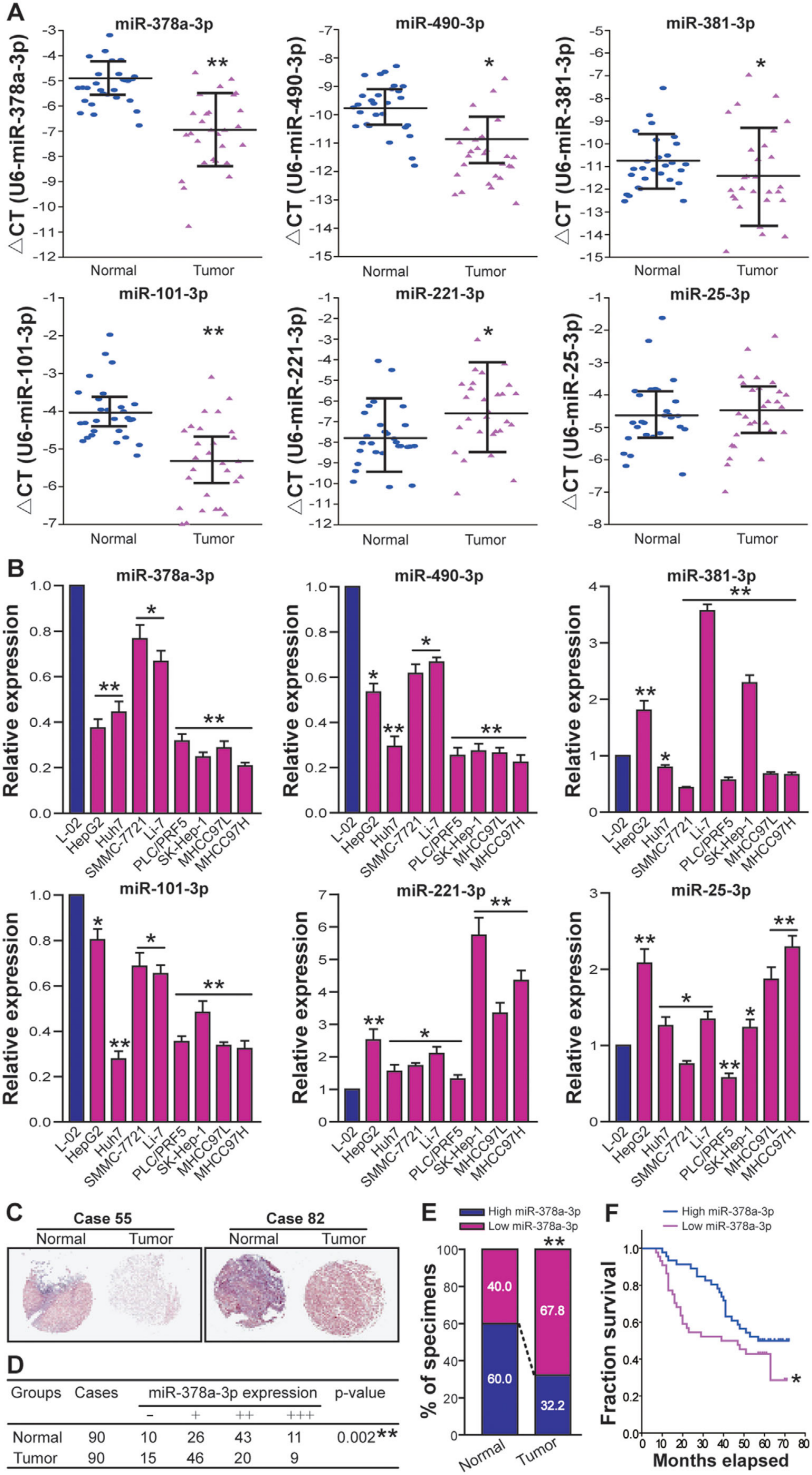
Verification of bioinformatics prediction of miRNAs in HCC and correlation of reduction of miR‐378a‐3p with poor prognosis in HCC patients. (A) RT‐qPCR analysis of miRNAs in HCC clinical tissue specimens. The expression levels of miR‐25‐3p, miR‐101‐3p, miR‐221‐3p, miR‐378a‐3p, miR‐381‐3p, and miR‐490‐3p in 28 pairs of human HCC tumor tissues (Tumor) and adjacent nontumor liver tissues (Normal) were normalized to U6 internal control and expressed as ∆*CT* = (mean *CT*_U6_ – mean *CT*_miRNA_). *p < 0.05 or **p < 0.01 compared with Normal, Student's *t* test, *n* = 6 replicates per sample. (B) RT‐qPCR analysis of miRNAs in HCC cell lines. The expression levels of miR‐25‐3p, miR‐101‐3p, miR‐221‐3p, miR‐378a‐3p, miR‐381‐3p, and miR‐490‐3p in a panel of human HCC cell lines including HepG2, HuH7, SMMC‐7721, Li‐7, PLC/PRF5, SK‐Hep‐1, MHCC97L, and MHCC97H were normalized to U6 internal control and calculated by a 2^−∆∆^ *^CT^* method, with 1 being the value for L‐02 normal live cell control. *p < 0.05 or **p < 0.01 compared with L‐02, Student's *t* test, *n* = 6 replicates per sample. (C) ISH analysis of miR‐378a‐3p in HCC TMA. The representative pictures of ISH (Case 55 T, –; and Case 82 T, ++) were shown. (D) Summary of miR‐378a‐3p ISH scoring. **p < 0.01 compared with Normal, Mann–Whitney *U* test, *n* = 90 cases. (E) The percentage of high and low miR‐378a‐3p expression in Tumor or Normal in HCC TMA. **p < 0.01 compared with Normal, Pearson's *χ* ^2^ test, *n* = 90 cases. (F) The relationship between expression level of miR‐378a‐3p and prognosis of HCC patients. *p < 0.05 compared with high miR‐378a‐3p expression, Log‐rank test

To verify the above‐described bioinformatical prediction of microRNAs’ expression pattern in HCC, the expression of miR‐25‐3p, miR‐101‐3p, miR‐221‐3p, miR‐378a‐3p, miR‐381‐3p, and miR‐490‐3p in 28 pairs of snap‐frozen human HCC tumor tissues and adjacent nontumor liver tissues (normal tissue control), as well as in eight human HCC cell lines (HepG2, HuH7, SMMC‐7721, Li‐7, PLC/PRF5, SK‐Hep‐1, MHCC97L, and MHCC97H) and a L‐02 human liver cell line (normal cell control), was determined by RT‐qPCR analysis. As shown in Figure 5A, compared to normal tissue control, the expressions of miR‐378a‐3p, miR‐490‐3p, miR‐381‐3p, and miR‐101‐3p were downregulated in HCC tumor tissues, whereas the expression of miR‐221‐3p was upregulated in HCC tumor tissues (*p* < 0.05 or < 0.01). Although no statistically significant difference was found in miR‐25‐3p, HCC tumor tissues still exhibited a slightly higher expression of miR‐25‐3p than normal tissues. Moreover, all eight tested HCC cell lines exhibited lower miR‐378a‐3p, miR‐490‐3p, and miR‐101‐3p expression and higher miR‐221‐3p expression, when compared with L‐02 normal liver cell control (*p *< 0.05 or < 0.01) (Figure 5B). The expression of miR‐381‐3p was found to be reduced in HuH7, SMMC‐7721, PLC/PRF5, MHCC97L, and MHCC97H HCC cell lines but elevated in HepG2, Li‐7, and SK‐Hep‐1 HCC cell lines (*p *< 0.05 or < 0.01) (Figure 5B). We also found higher level of miR‐25‐3p in HepG2, HuH7, Li‐7, SK‐Hep‐1, MHCC97L, and MHCC97H HCC cell lines, but lower level of miR‐25‐3p in SMMC‐7721 and PLC/PRF5 HCC cell lines (*p *< 0.05 or < 0.01) (Figure 5B). Thus, the expression trend of miR‐25‐3p, miR‐101‐3p, miR‐221‐3p, miR‐378a‐3p, miR‐381‐3p, and miR‐490‐3p in human HCC clinical tissue specimens and cell lines was largely consistent with our bioinformatical prediction.

### 3.8 MiR‐378a‐3p reduction correlates with clinicopathological features and poor prognosis of HCC

To further examine whether downregulation of miR‐378a‐3p is clinically relevant in HCC, we extended HCC cases and determined the expression of miR‐378a‐3p in HCC TMA (90 cases, 90 paired human HCC, and adjacent normal tissues) by ISH analysis (Figure 5C). As shown in Figures 5D and 5E, low expression of miR‐378a‐3p in tumor tissues occurred in 61 cases (15 cases scored “–” and 46 cases scored “+”) (67.8%), whereas high expression of miR‐378a‐3p only occurred in 29 cases (20 cases scored “++” and nine cases scored “+++”) (32.2%). Among matched adjacent normal tissues, 54 cases (60.0%) displayed high expression of miR‐378a‐3p (43 cases scored “++” and 11 cases scored “+++”), whereas 36 cases (40.0%) exhibited low expression of miR‐378a‐3p (10 case scored “–” and 26 cases scored “+”). Our results demonstrated a decreased expression of miR‐378a‐3p in HCC tumor tissues compared to adjacent normal tissues (*p *< 0.01). Based on miR‐378a‐3p expression in HCC tumor tissues, 90 patients with HCC were divided into two groups: low–expression group (“–” or “+”, *n* = 61) and high–expression group (“++” or “+++”, *n* = 29). To evaluate the clinical significance of miR‐378a‐3p in HCC, the correlation between miR‐378a‐3p expression in human HCC tissues and HCC clinicopathological features was analyzed. As shown in Table 3, miR‐378a‐3p expression was inversely correlated with tumor size, vascular invasion, and TNM stage (*p *< 0.05). A Kaplan–Meier survival analysis further confirmed that low level of miR‐378a‐3p led to a significant reduction in the overall survival rate of HCC patients (*p *< 0.05) (Figure 5F). Moreover, similar survival patterns were noticed during the analysis of patient data from The Cancer Genome Atlas (TCGA) (File S4). Our data indicated that miR‐378a‐3p reduction may facilitate human HCC progression, and it can therefore be used as a prognostic indicator for HCC.

**TABLE 3:** The relationship of miR‐378a‐3p expression with HCC clinicopathological features

Variables	High miR‐378a‐3p expression (*n* = 29)	Low miR‐378a‐3p expression (*n* = 61)	*p*‐Value
Gender			0.8436
Female	13	26	
Male	16	35	
Age (years)			0.7965
≤60	12	27	
>60	17	34	
HbsAg			0.7497
Negative	9	21	
Positive	20	40	
Cirrhosis			0.8977
Absent	11	24	
Present	18	37	
Serum AFP (ng/mL)			0.8834
≤20	10	22	
>20	19	39	
Tumor size (cm)			0.0289*
≤5	16	19	
>5	13	42	
Tumor number			0.8509
Single	21	43	
Multiple	8	19	
Vascular invasion			0.0410*
Absent	15	18	
Present	14	43	
TNM stage			0.0199*
Early (I/II)	17	20	
Late (III/IV)	12	41	

* *p* < 0.05, Pearson's *χ* ^2^ test.

Abbreviations: AFP, alpha‐fetoprotein; TNM, A system for cancer staging. T indicates the size of the tumor and any spread of cancer into nearby tissue; N indicates spread of cancer to nearby lymph nodes; and M indicates metastasis.

### 3.9 MiR‐378a‐3p inhibits HCC cell growth in vitro and in vivo

**FIGURE 6:**
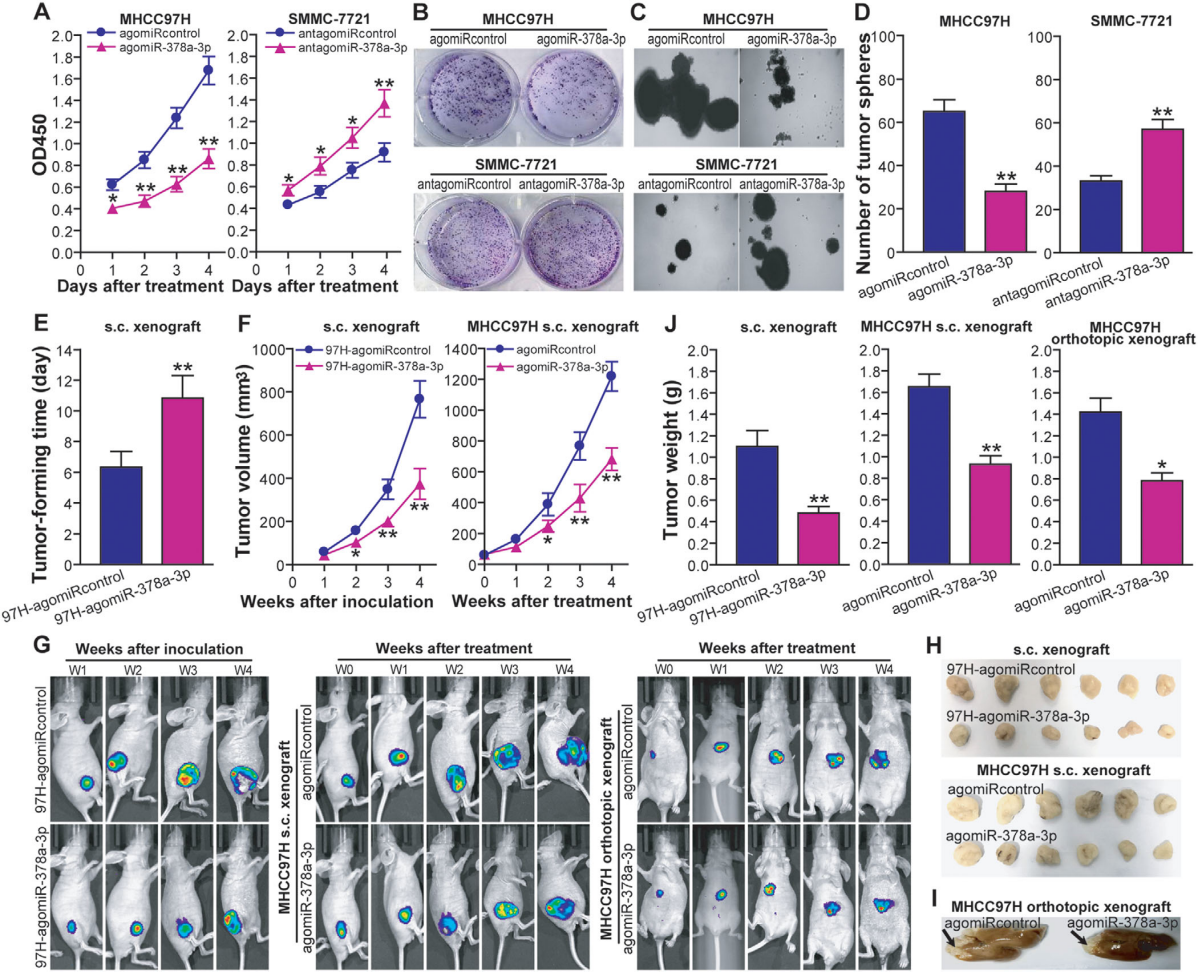
MiR‐378a‐3p suppresses HCC cell proliferation and growth *in vitro* and *in vivo* in athymic nude mice. (A) CCK‐8 assay. MHCC97H: *p < 0.05 at day 1 or **p < 0.01 at day 2, 3, and 4 after treatment with agomiR‐378a‐3p, compared with MHCC97H‐agomiRcontrol; SMMC‐7721: *p < 0.05 at day 1, 2, and 3 or **p < 0.01 at day 4 after treatment with antagomiR‐378a‐3p, compared with SMMC‐7721‐antagomiRcontrol, two‐way repeated measures analysis of variance (ANOVA), *n* = 6 replicates per condition. (B) Colony formation assay. The representative pictures of colonies were shown. (C and D) Microsphere‐forming assay. The representative pictures of tumor microspheres (C) were shown and the number of tumor microspheres (D) was counted. MHCC97H: **p < 0.01 compared with MHCC97H‐agomiRcontrol; SMMC‐7721: **p < 0.01 compared with SMMC‐7721‐antagomiRcontrol, Student's *t* test, *n* = 6 replicates per condition. (E) Tumor‐forming time. **p < 0.01 compared with MHCC97H‐agomiRcontrol, Student's *t* test, *n* = 6 replicates per condition. (F) Tumor volume. Tumor (s.c.) formation assay (left): *p < 0.05 at week 2 or **p < 0.01 at weeks 3 and 4 after inoculation of MHCC97H‐agomiR‐378a‐3p tumor cells, compared with MHCC97H‐agomiRcontrol; tumor (s.c.) treatment assay (right): *p < 0.05 at week 2 or **p < 0.01 at weeks 3 and 4 after treatment of MHCC97H s.c. xenografted tumors with agomiR‐378a‐3p, compared with agomiRcontrol treatment, two‐way repeated measures ANOVA, *n* = 6 replicates per condition. (G) In vivo luciferase tumor imaging. The representative pictures of in vivo tumor imaging were shown. Tumor (s.c.) formation assay (left); tumor (s.c.) treatment assay (middle); tumor (orthotopic) treatment assay (right). (H) The photos of s.c. xenografted tumors. Tumor (s.c.) formation assay (Upper); tumor (s.c.) treatment assay (lower). (I) The representative photos of orthotopically xenografted tumors (marked with arrows). (J) Tumor weight. Tumor (s.c.) formation assay (left): **p < 0.01 compared with MHCC97H‐agomiRcontrol; tumor (s.c.) treatment assay (middle): **p < 0.01 compared with agomiRcontrol treatment of MHCC97H s.c. xenografted tumors; tumor (orthotopic) treatment assay (right): *p < 0.05 compared with agomiRcontrol treatment of MHCC97H orthotopically xenografted tumors, Student's *t* test, *n* = 6 replicates per condition

To examine the effect of miR‐378a‐3p on human HCC cell growth, the in vitro proliferation abilities of MHCC97H HCC cells treated with agomiR‐378a‐3p or agomiRcontrol, as well as those of SMMC‐7721 HCC cells treated with antagomiR‐378a‐3p or antagomiRcontrol, were determined by a CCK‐8 assay. As shown in Figure 6A, the growth in the MHCC97H‐agomiR‐378a‐3p was much slower than that in the MHCC97H‐agomiRcontrol control (*p *< 0.05 or < 0.01), demonstrating that miR‐378a‐3p inhibits MHCC97H tumor cell growth in vitro. In contrast, inhibition of miR‐378a‐3p using antagomiR‐378a‐3p significantly promoted SMMC‐7721 tumor cell growth compared with SMMC‐7721‐antagomiRcontrol control (*p *< 0.05 or < 0.01) (Figure 6A). Consistently, MHCC97H‐agomiR‐378a‐3p tumor cells formed smaller and less colonies (Figure 6B) or tumor microspheres (Figures 6C and 6D) than MHCC97H‐agomiRcontrol control cells (*p *< 0.01). The colonies (Figure 5B) or tumor microspheres (Figures 6C and 6D) that grew from SMMC‐7721‐antagomiR‐378a‐3p tumor cells were larger in size and greater in quantity than those from SMMC‐7721‐antagomiRcontrol control cells (*p* < 0.01). Our data indicated that miR‐378a‐3p also suppresses clonogenicity and self‐renewal activity of HCC cells. To further determine whether the correlation between miR‐378a‐3p and HCC growth traits in vitro could be reproduced in vivo, we monitored human HCC s.c. xenografted tumor growth of MHCC97H tumor cells pretreated with agomiR‐378a‐3p or agomiRcontrol in athymic BALB/c nude mice. As shown in Figures 6E–H and 6J, miR‐378a‐3p suppressed MHCC97H tumor cell growth in vivo (*p *< 0.05 or < 0.01). In the other animal studies, the athymic BALB/c nude mice were s.c. or orthotopically implanted with MHCC97H tumor cells to establish HCC s.c. or orthotopically xenografted tumors, and then subjected to treatment with agomiR‐378a‐3p or agomiRcontrol. We found that miR‐378a‐3p treatment significantly inhibited pre‐established MHCC97H s.c. (Figures 6F–H and 6J) and orthotopic (Figures 6G, 6I, and 6J) tumor growth in athymic nude mice (*p* < 0.05 or < 0.01). Taken together, our data demonstrated the HCC cell growth repression ability of miR‐378a‐3p both in vitro and in vivo.

### 3.10 MiR‐378a‐3p suppresses HCC cell migration, invasion, and distant lung metastasis

**FIGURE 7:**
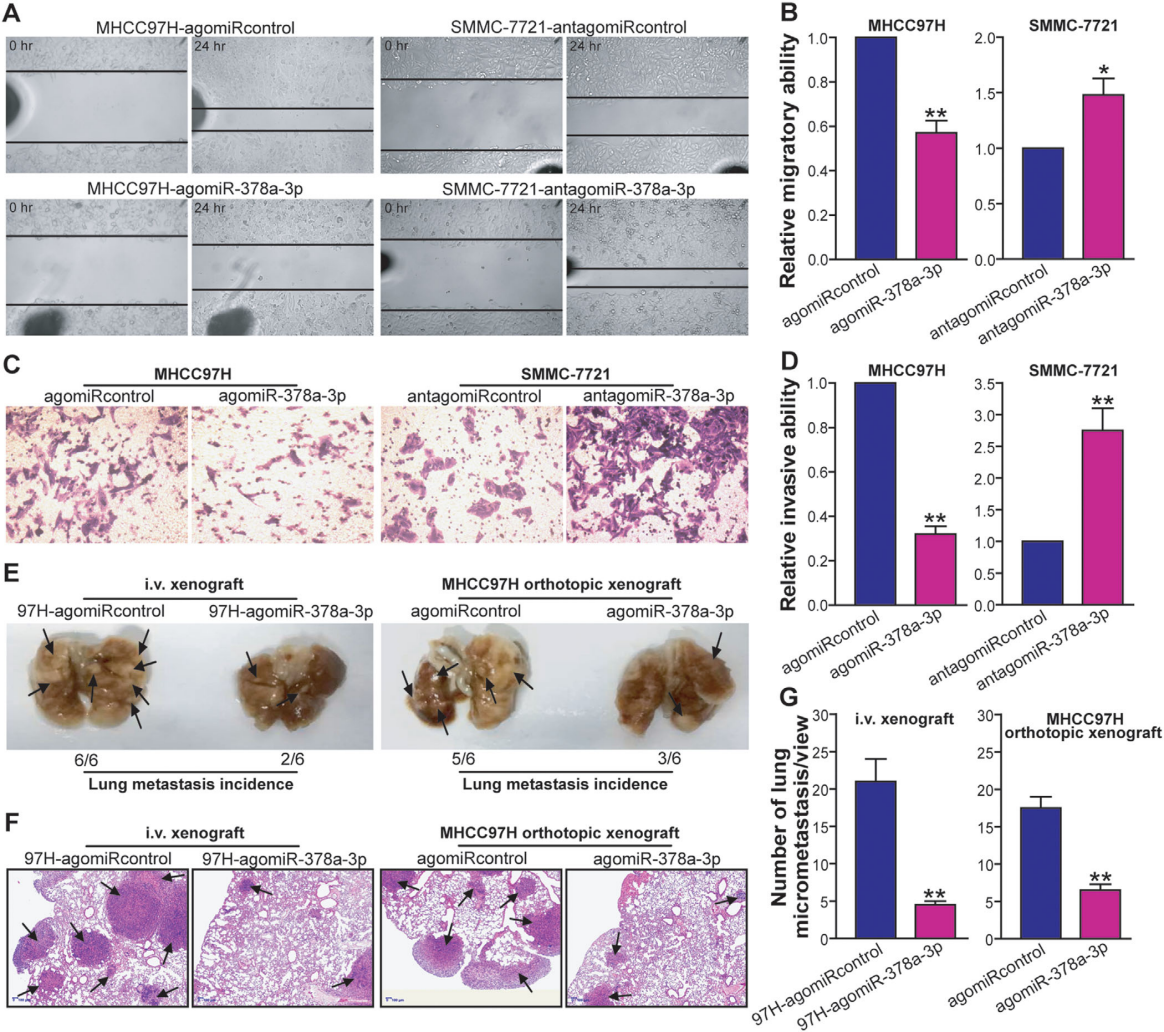
MiR‐378a‐3p inhibits HCC cell migration and invasion in vitro and lung metastasis in vivo. Scratch assay. The representative photos of scratch assay of the indicated cells were shown in panel A. (B) The relative migratory ability was calculated and expressed as a ratio or fold of MHCC97H‐agomiRcontrol or SMMC‐7721‐antagomiRcontrol control, with 1 being the value for controls. MHCC97H: **p < 0.01 compared with MHCC97H‐agomiRcontrol; SMMC‐7721: *p < 0.05 compared with SMMC‐7721‐antagomiRcontrol, Student's *t* test, *n* = 6 replicates per condition. (C) Transwell invasion assay. The representative photos of Transwell invasion assay of the indicated cells were shown. (D) The relative invasive ability was calculated. MHCC97H: **p < 0.01 compared with MHCC97H‐agomiRcontrol; SMMC‐7721: **p < 0.01 compared with SMMC‐7721‐antagomiRcontrol, Student's *t* test, *n* = 3 replicates per condition, *n* = 5 observations per replicate. (E–G) In vivo lung metastasis assay. The representative photos of lung tissue specimen (E) and lung tissue HE staining (F) were shown. The tumor metastasis nodules in the lungs were marked with arrows (E and F). Tumor (i.v.) injection assay (left); tumor (orthotopic) treatment assay (right). The tumor micrometastasis nodules in the lungs were counted according to H&E staining (G). Tumor (i.v.) injection assay (left): **p < 0.01 compared with MHCC97H‐agomiRcontrol; tumor (orthotopic) treatment assay (right): **p < 0.01 compared with agomiRcontrol treatment of MHCC97H orthotopically xenografted tumors, Student's *t* test, *n* = 6 replicates per condition, *n* = 5 sections per sample, *n* = 5 observations per section

To analyze the association of miR‐378a‐3p with metastatic potential of HCC cells, scratch assay and Transwell invasion assay were conducted to examine the in vitro migratory and invasive abilities of MHCC97H‐agomiR‐378a‐3p versus MHCC97H‐agomiRcontrol HCC cells, as well as SMMC‐7721‐antagomiR‐378a‐3p versus SMMC‐7721‐antagomiRcontrol HCC cells, respectively. As shown in Figures 7A and 7B, the migratory capacity of MHCC97H‐agomiR‐378a‐3p tumor cells was dramatically impaired compared with MHCC97H‐agomiRcontrol control cells (*p *< 0.01). Inhibition of miR‐378a‐3p enhanced the migratory ability in SMMC‐7721 tumor cells (*p *< 0.05) (Figures 7A and 7B). Furthermore, miR‐378a‐3p suppressed MHCC97H tumor cell invasion, whereas antagonism of miR‐378a‐3p promoted the process in SMMC‐7721 tumor cells (*p *< 0.01) (Figures 7C and 7D). To further investigate whether the correlation between miR‐378a‐3p and HCC metastatic traits in vitro could be reproduced in vivo, the MHCC97H‐agomiR‐378a‐3p and MHCC97H‐agomiRcontrol tumor cells were injected into the tail vein of athymic BALB/c nude mice, respectively. Four weeks after the intravenous injection, the lung tissues of the mice were removed. As shown in Figure 7E, those mice injected with MHCC97H‐agomiR‐378a‐3p tumor cells exhibited a significant decrease in tumor lung metastasis nodule than those injected with MHCC97H‐agomiRcontrol control cells. Moreover, the pulmonary metastasis frequency (2/6) in the MHCC97H‐agomiR‐378a‐3p‐injected mice was lower than that (6/6) in the MHCC97H‐agomiRcontrol control‐injected mice. The tumor lung micrometastasis nodules were further examined and counted by HE histological analysis, which showed a significant reduction in in MHCC97H‐agomiR‐378a‐3p‐injected mice (*p *< 0.01) (Figures 7F and 7G). In addition, miR‐378a‐3p treatment using agomiR‐378a‐3p also suppressed the distant lung metastasis of MHCC97H orthotopic xenograft tumors (*p *< 0.01) (Figure 7E–G). These data indicated that miR‐378a‐3p efficiently weakens the metastasis potential of HCC cells.

### 3.11 MiR‐378a‐3p represses PLAGL2 expression in HCC cells

**FIGURE 8:**
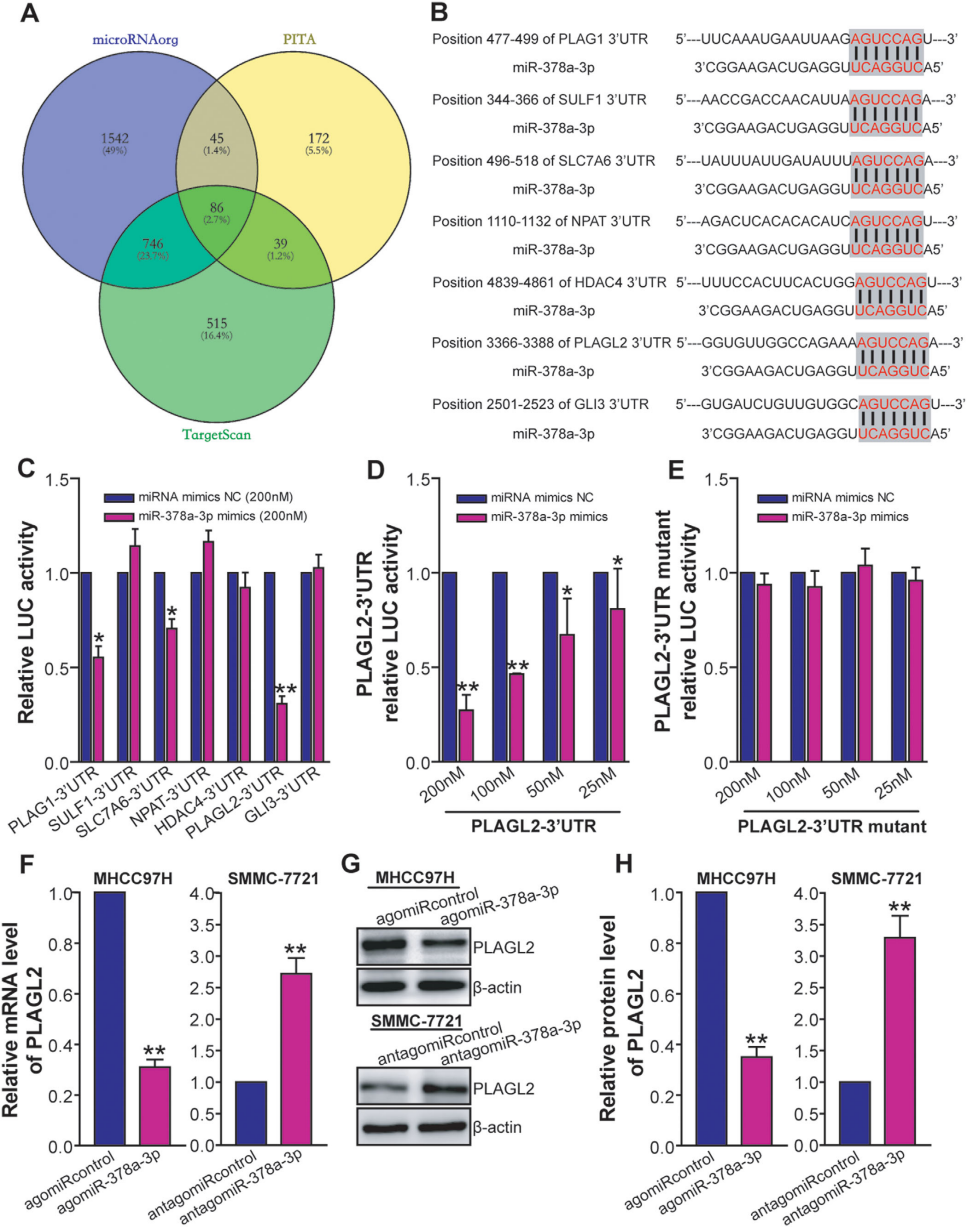
MiR‐378a‐3p suppresses PLAGL2 expression in HCC cells. (A) Heatmap of the predicted targets for miR‐378a‐3p. (B) Schematic miR‐378a‐3p putative target sites in 3′UTRs of PLAG1, SULF1, SLC7A6, NPAT, HDAC4, PLAGL2, and GLI3. (C–E) 3′UTR‐dual luciferase reporter assays. The wild‐type PLAG1‐3′UTR, SULF1‐3′UTR, SLC7A6‐3′UTR, NPAT‐3′UTR, HDAC4‐3′UTR, PLAGL2‐3′UTR, or GLI3‐3′UTR reporter plasmid was cotransfected with 200 nM of miR‐378a‐3p mimics or miRNA mimics NC into 293T cells (C). The wild‐type (D) or mutant (E) PLAGL2‐3′UTR reporter plasmid was cotransfected with various amounts (25, 50, 100, and 200 nM) of miR‐378a‐3p mimics or miRNA mimics NC into 293T cells. The relative luciferase activity was expressed as a ratio or fold of miRNA mimics NC cotransfection control, with 1 being the value for control. *p < 0.05 or **p < 0.01 compared with miRNA mimics NC, Student's *t* test, *n* = 3 replicates per condition, *n* = 3 replicates per sample. (F) RT‐qPCR analysis of PLAGL2. The mRNA level in MHCC97H‐agomiR‐378a‐3p or SMMC‐7721‐antagomiR‐378a‐3p was normalized to β‐actin internal control and calculated by a 2^−∆∆^ *^CT^* method, with 1 being the value for MHCC97H‐agomiRcontrol or SMMC‐7721‐antagomiRcontrol control. MHCC97H: **p < 0.01 compared with MHCC97H‐agomiRcontrol; SMMC‐7721: **p < 0.01 compared with SMMC‐7721‐antagomiRcontrol, Student's *t* test, *n* = 3 replicates per condition, *n* = 3 replicates per sample. (G and H) Western blot analysis of PLAGL2. The representative pictures of Western blot were shown (G). The protein level of PLAGL2 in MHCC97H‐agomiR‐378a‐3p or SMMC‐7721‐antagomiR‐378a‐3p was normalized to β‐actin internal control (PLAGL2/β‐actin) and expressed as a ratio or fold of respective control, with 1 being the value for MHCC97H‐agomiRcontrol or SMMC‐7721‐antagomiRcontrol control (H). MHCC97H: **p < 0.01 compared with MHCC97H‐agomiRcontrol; SMMC‐7721: **p < 0.01 compared with SMMC‐7721‐antagomiRcontrol, Student's *t* test, *n* = 3 replicates per condition, *n* = 3 replicates per sample

To explore the mechanism by which miR‐378a‐3p suppresses growth and metastasis of HCC cells, the biological targets of miR‐378a‐3p were predicted by microRNAorg, PITA, and TargetScan databases (Figure 8A). Among the predicted targets, seven potential target genes of miR‐378a‐3p including PLAG1, SULF1, SLC7A6, NPAT, HDAC4, PLAGL2, and GLI3 were selected. We further confirmed that PLAG1, SULF1, SLC7A6, NPAT, HDAC4, PLAGL2, and GLI3 were putative targets of miR‐378a‐3p using bioinformatics tools (Figure 8B). 3′UTR‐dual luciferase reporter assay showed that the expression of the wild‐type PLAG1, SLC7A6, or PLAGL2, especially PLAGL2, was inhibited in miR‐378a‐3p mimics‐transfected 293T cells compared to miRNA mimics NC‐transfected control cells (*p *< 0.05 or < 0.01) (Figure 8C). Moreover, miR‐378a‐3p mimics repressed expression of wild‐type PLAGL2 in a dose‐dependent manner (*p *< 0.05 or < 0.01) (Figure 8D). Importantly, miR‐378a‐3p mimics failed to suppress the expression of the mutant 3′UTR of PLAGL2 (Figure 8E). RT‐qPCR (Figure 8F) and western blot (Figures 8G and 8H) analyses further showed that overexpression of miR‐378a‐3p with agomiR‐378a‐3p downregulated both the mRNA and protein levels of PLAGL2 in MHCC97H tumor cells, whereas inhibition of miR‐378a‐3p with antagomiR‐378a‐3p upregulated the levels of PLAGL2 in SMMC‐7721 tumor cells (*p *< 0.01). Collectively, our data indicated that PLAGL2 is a functional target of miR‐378a‐3p, and miR‐378a‐3p directly suppresses PLAGL2 expression in HCC cell lines.

### 3.12 MiR‐378a‐3p inhibits PLAGL2/β‐catenin signaling

**FIGURE 9:**
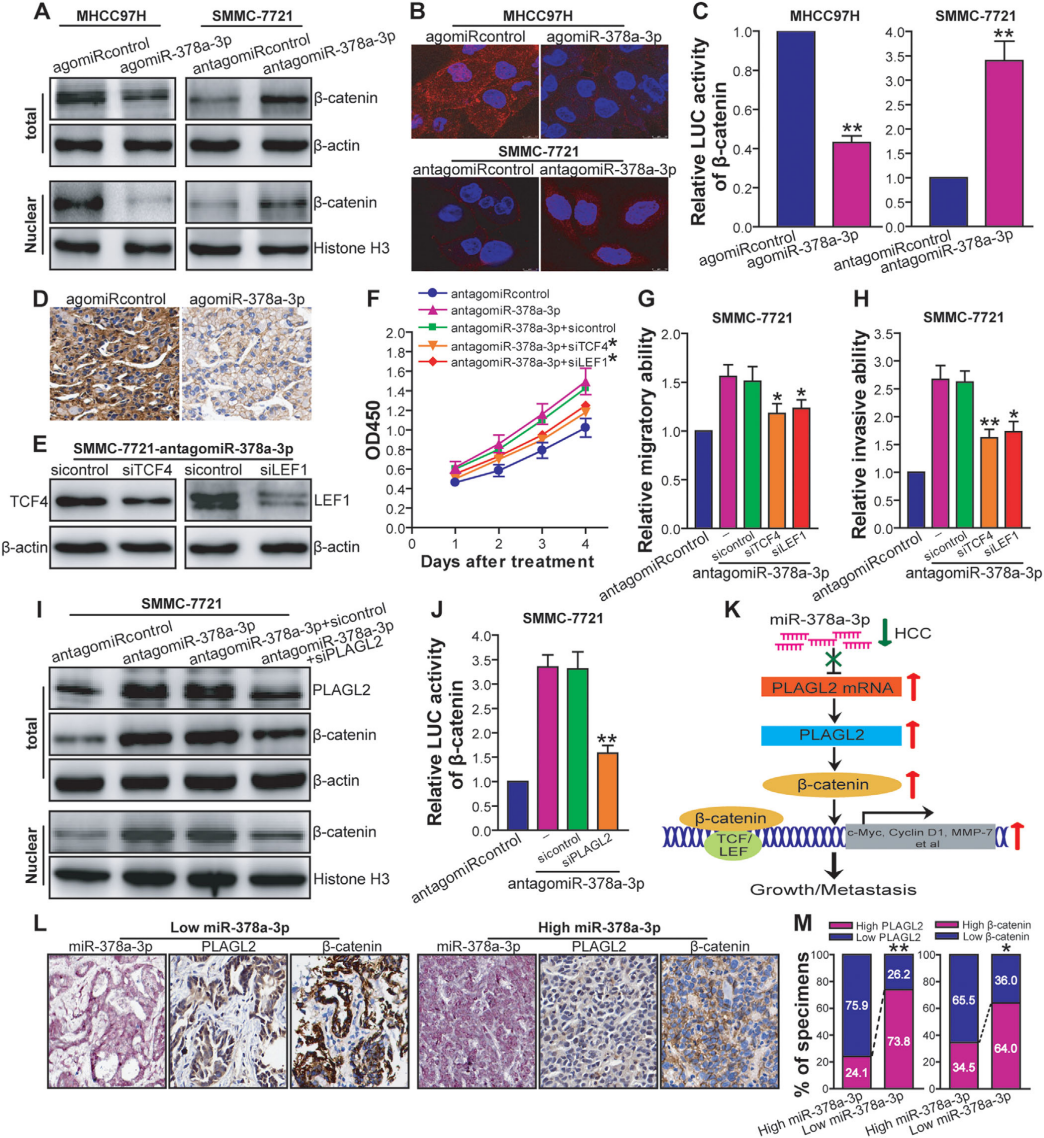
MiR‐378a‐3p inhibits HCC cell growth and metastasis via suppressing PLAGL2/β‐catenin signaling. (A) Western blot analysis of β‐catenin. The representative pictures of Western blot were shown. (B) Immunofluorescence confocal microscopic analysis of β‐catenin. The representative pictures of merge (red, β‐catenin‐Alexa Fluor 647; blue, 4′,6‐diamidino‐2‐phenylindole [DAPI]) were shown. (C) Luciferase reporter analysis of transcriptional activity of β‐catenin. The relative luciferase activity was expressed as a ratio or fold of MHCC97H‐agomiRcontrol or SMMC‐7721‐antagomiRcontrol control, with 1 being the value for controls. MHCC97H: **p < 0.01 compared with MHCC97H‐agomiRcontrol; SMMC‐7721: **p < 0.01 compared with SMMC‐7721‐antagomiRcontrol, Student's *t* test, *n* = 3 replicates per condition, *n* = 3 replicates per sample. (D) Immunohistochemistry analysis of β‐catenin in HCC orthotopically xenografted tumors. The representative pictures of immunohistochemistry were shown. (E) Western blot analysis of TCF4 or LEF1 siRNA‐mediated knockdown of TCF4 or LEF1. The representative pictures of Western blot were shown. (F) CCK‐8 assay after TCF4 or LEF1 knockdown. *p < 0.05 compared with SMMC‐7721‐antagomiR‐378a‐3p or control siRNA‐transfected SMMC‐7721‐antagomiR‐378a‐3p at day 1, 2, 3, and 4, two‐way repeated measures analysis of variance (ANOVA), *n* = 6 replicates per condition. (G) Scratch assay after TCF4 or LEF1 knockdown. *p < 0.05 compared with SMMC‐7721‐antagomiR‐378a‐3p or control siRNA‐transfected SMMC‐7721‐antagomiR‐378a‐3p, one‐way repeated measures ANOVA, *n* = 6 replicates per condition. (H) Transwell invasion assay after TCF4 or LEF1 knockdown. TCF4 siRNA: **p < 0.01; LEF1 siRNA: *p < 0.05 compared with SMMC‐7721‐antagomiR‐378a‐3p or control siRNA‐transfected SMMC‐7721‐antagomiR‐378a‐3p, one‐way repeated measures ANOVA, *n* = 6 replicates per condition. (I) Western blot analysis of PLAGL2 siRNA‐mediated PLAGL2 knockdown as well as β‐catenin after PLAGL2 knockdown. The representative pictures of Western blot were shown. (J) Luciferase reporter analysis of transcriptional activity of β‐catenin after PLAGL2 knockdown. **p < 0.01 compared with SMMC‐7721‐antagomiR‐378a‐3p or control siRNA‐transfected SMMC‐7721‐antagomiR‐378a‐3p, one‐way repeated measures ANOVA, *n* = 3 replicates per condition, *n* = 3 replicates per sample. (K) A schematic model of miR‐378a‐3p's function during HCC growth and metastasis. (L and M) Clinical relevance of miR‐378a‐3p with PLAGL2 and β‐catenin in HCC. (L) The representative pictures of ISH analysis of miR‐378a‐3p as well as immunohistochemistry analysis of PLAGL2 and β‐catenin in HCC tissue specimens derived from two representative cases (Case 15, +, low miR‐378a‐3p; and Case 17, +++, high miR‐378a‐3p) were shown. (M) The percentage of specimens exhibiting high or low miR‐378a‐3p expression in relation to the expression levels of PLAGL2 and β‐catenin was shown. PLAGL2: **p < 0.01; β‐catenin: *p < 0.05, Pearson's *χ* ^2^ test

Previous studies showed that PLAGL2 can elevate Wnt/β‐catenin signaling in some type of human cancers.^45^, ^46^ To address whether miR‐378a‐3p‐mediated PLAGL2 downregulation would lead to inactivation of β‐catenin signaling in human HCC cells, we analyzed the expression, subcellular location, and transcriptional activity of β‐catenin by western blot, confocal microscopy, and luciferase reporter analysis, respectively. As shown in Figure 9A, both the total and nuclear levels of β‐catenin were markedly reduced in MHCC97H‐agomiR‐378a‐3p tumor cells compared to MHCC97H‐agomiRcontrol control cells. Conversely, inhibition of miR‐378a‐3p increased the total and nuclear levels of β‐catenin in SMMC‐7721 tumor cells. Confocal microscopic analysis also showed that miR‐378a‐3p overexpression impeded accumulation and translocation of β‐catenin to nucleus in MHCC97H tumor cells, whereas inhibition of miR‐378a‐3p exhibited an opposing effect in SMMC‐7721 tumor cells (Figure 9B). Of note, miR‐378a‐3p suppressed the transcriptional activity of β‐catenin in MHCC97H tumor cells, whereas antagonism of miR‐378a‐3p enhanced it in SMMC‐7721 tumor cells (*p *< 0.01) (Figure 9C). The in vivo downregulatory effect of miR‐378a‐3p on β‐catenin was further confirmed by IHC analysis of β‐catenin in agomiR‐378a‐3p‐pretreated or agomiR‐378a‐3p‐treated MHCC97H s.c. xenografted tumors (data not shown) and agomiR‐378a‐3p‐treated MHCC97H orthotopically xenografted tumors (Figure 9D). To check whether β‐catenin is involved in miR‐378a‐3p‐elicited functional effects in HCC, we further analyzed the effect of blocking β‐catenin signaling via siRNA‐mediated knockdown of TCF4 or LEF1 (Figure 9E) on the proliferative, migratory, and invasive capacity of SMMC‐7721‐antagomiR‐378a‐3p tumor cells. As shown in Figure 9F–H, inhibition of β‐catenin signaling remarkably attenuated the miR‐378a‐3p antagonism‐induced proliferation, migration, and invasion in SMMC‐7721 tumor cells (*p *< 0.01 or < 0.05). Our results indicated that Wnt/β‐catenin signaling is a functional mediator for miR‐378a‐3p reduction‐induced growth and metastasis in HCC cells. To further explore the functional significance of PLAGL2 in β‐catenin activation induced by miR‐378a‐3p downregulation in human HCC cells, we studied the effect of siRNA‐mediated PLAGL2 knockdown on β‐catenin signaling in SMMC‐7721‐antagomiR‐378a‐3p tumor cells. We found that knockdown of PLAGL2 significantly abrogated miR‐378a‐3p antagonism‐induced enhancement of β‐catenin signaling in SMMC‐7721 tumor cells (*p *< 0.01) (Figures 9I and 9J), suggesting that miR‐378a‐3p reduction‐triggered upregulation of PLAGL2 is functionally important for activation of β‐catenin in HCC cells. Taken together, these results indicated that miR‐378a‐3p suppresses HCC growth and metastasis via inhibition of PLAGL2/β‐catenin signaling. The reduction or loss of miR‐378a‐3p in HCC may upregulate PLAGL2 expression and β‐catenin signaling, leading to the progression of HCC (Figure 9K).

### 3.13 Clinical association between miR‐378a‐3p and PLAGL2/β‐catenin in HCC

To confirm the regulatory relationship between miR‐378a‐3p and its target, PLAGL2, or β‐catenin in human HCC clinical tissue specimens, the expression of miR‐378a‐3p and PLAGL2/β‐catenin in HCC TMA (90 cases) was analyzed by ISH and IHC, respectively. As shown in Figure 9L, the immunostaining intensity of PLAGL2 and total/nuclear β‐catenin was much stronger in HCC tumor tissues with low miR‐378a‐3p expression than in those with high miR‐378a‐3p expression. Furthermore, 73.8% of cases with low miR‐378a‐3p expression (45/61 cases) in HCC tumor tissues showed a high level of PLAGL2, whereas only 24.1% of cases with high miR‐378a‐3p expression (7/29 cases) exhibited a high level of PLAGL2 (*p *< 0.01) (Figure 9M). Also, HCC cases with low miR‐378a‐3p expression displayed a higher level of β‐catenin (39 of 61 cases; 64.0%) than ones with high miR‐378a‐3p expression (10 of 29 cases; 34.5%) (*p *< 0.05) (Figure 9M). Consistent with the cell model results, clinical data also demonstrated that miR‐378a‐3p expression is inversely correlated with the expression pattern of PLAGL2 and β‐catenin and the activation of β‐catenin signaling.

## 4 DISCUSSION

In this study, we integrated cancer gene expression data and network model of microRNA–mRNA interactions by calculating NOD and TFP values to find the key microRNA biomarkers in HCC. Bioinformatics methods were used to predict targets of microRNAs, and enrichment analyses were performed on these target genes. Subsequently, these results were verified by experimental methods both in vivo and in vitro experiments. We found that miR‐378a‐3p acted as a tumor‐suppressor gene in HCC. Abnormal expression of miR‐378a‐3p impacted on tumor cell growth and invasion, which could help researchers develop an early diagnostic biomarker for HCC. However, it is still necessary to conduct both biological and clinical investigations to further explore this topic.

Among six candidate microRNA biomarkers predicted by our model, miR‐25‐3p, miR‐221‐3p, and miR‐101‐3p have been reported to have prognostic value for HCC. MiR‐25 (miR‐25‐3p) is highly expressed and is associated with poor prognosis in HCC tissues.^47^ MiR‐25‐3p is also significantly elevated in HCC plasma and can be used in combination with other seven microRNAs to distinguish between HCC patients and noncancer controls.^48^ MiR‐221‐3p plays an important role in the tumor formation. It stimulates cellular growth and proliferation by targeting cell cycle inhibitors, cyclin‐dependent kinase inhibitor 1C (p57) and 1B (p27).^49^, ^50^ It also inhibits cell death by regulating Bcl‐2‐modifying factor.^51^ Moreover, miR‐221‐3p is overexpressed in HCC tissues and serum,^50^, ^52^, ^53^ and the high expression level of MiR‐221‐3p in serum is associated with tumor size, tumor stage, and poor prognosis.^54^, ^55^ MiR‐101‐3p is significantly downregulated in HCC samples and suppresses tumor, thus a potential biomarker in tumorigenesis.^56^, ^57^, ^58^, ^59^ High miR‐101‐3p expression can inhibit the expression of myeloid cell leukemia‐1 (Mcl‐1) and promote apoptosis.^60^ The ectopic miR‐101 expression can imitate the inhibitory effect of nemo‐like kinase on HCC, repress cancer cell growth and proliferation,^61^ and inhibit the development of HCC by reducing the expression of EZH2.^62^ Downregulation of miR‐101‐3p is associated with invasiveness and poor prognosis of HCC,^63^ and low expression of plasma miR‐101‐3p can predict a worse disease‐free survival.^64^

In addition, although there is no direct evidence that miR‐378a‐3p, miR‐490‐3p, and miR‐381‐3p could act as potential biomarkers for HCC, they also play key roles in the occurrence and development of HCC. A genetic variant, rs1076064, in miR‐378a‐3p precursor RNA is positively correlated to hepatitis B virus HCC risk and prognosis.^65^ The expression level of miR‐378 in HCC blood and tissues of patients is significantly lower than that in the control group,^66^, ^67^ and reduced expression of miR‐378 is associated with promoter hypermethylation. MiR‐490‐3p also shows significant downregulation in HCC tissue samples,^14^ and it modulates HCC cell growth and epithelial–mesenchymal transition by targeting endoplasmic reticulum–Golgi intermediate compartment protein 3.^68^ Similarly, miR‐381‐3p is also significantly underexpressed in HCC tissues and cell lines, whereas overexpression of miR‐381‐3p significantly suppresses HCC cell proliferation and invasion, and induces G0/G1 cell cycle arrest by directly targeting liver receptor homolog‐1.^69^ In summary, additional studies are required to explore the potentials of these microRNAs as biomarkers of HCC.

Our work integrated a rational microRNA biomarker discovery model and experimental functional analysis to yield valuable research results. For translation to clinical application, further researches are warranted. First, the occurrence and development of diseases is extremely heterogeneous due to the differences of genetics, living habits, occupations, and living environments in the population. As a result, finding universal biomarkers requires large sample size to validate their good application prospects and effectiveness. Second, to identify population‐personalized biomarkers, a personalized medicine model depends on more accurate informationization support. Finally, due to the complexity of diseases, it is only possible to conduct rational drug design or treatment from the perspective of system control and dynamics to interfere with disease development based on the in‐depth analysis of the molecular mechanism.

Besides, there also remain limitations in this study. First, we noticed that the heterogeneity among patients has significant influence on the evaluation of candidate biomarkers. Thus, a large‐scale analysis of patient microarray is demanded in the future to enhance the robustness and lower the inaccuracy of candidate biomarkers. What's more, other data, such as real‐world patient health records and multiple‐level omics data, are also need to be integrated to further describe the biomedical function of miR‐378 and explain the necessity as a biomarker for HCC.

In conclusion, we introduced a bioinformatics model that integrated network topological and functional evidence to identify microRNA biomarkers in HCC diagnosis. The predictions from our prosed model were further validated by experimental methods using human HCC cell lines, model animal, and clinical specimens. Notably, we found that miR‐378a‐3p was a tumor suppressor and its abnormal expression could affect the cell growth and invasion of HCC, which had both theoretical and clinical significance.

## AUTHOR CONTRIBUTIONS

SBR, XJ, YX, and FQ conceived and designed the project. JW, YW, and XJ provided the clinical samples. FQ, WY, and YL collected the data and performed the computational analyses. FQ and YX designed the experiments and performed the research. QFL, YX, and BS analyzed and interpreted data. QFL, YW, LS, and BS wrote the paper. All the authors read and approved the final manuscript.

## CONFLICT OF INTEREST

The authors declare no conflict of interest.

## Supporting information

Supporting Information

Supporting Information

Supporting Information

Supporting Information

## Data Availability

The data that support the findings of this study are partly available from GEO dataset: GSE63046, GSE21279, and GSE36915, the rest of the data are available from the corresponding author upon reasonable request.
