# Supplementary material for: MiR‐378a‐3p as a putative biomarker for hepatocellular carcinoma diagnosis and prognosis: Computational screening with experimental validation
Source: Clin Transl Med. 2021 Feb 14;11(2):e307. doi: 10.1002/ctm2.307 (PMC7882078; doi:10.1002/ctm2.307)
Supplement: Supplementary file 1 — Supporting Information [file CTM2-11-e307-s001.doc]

**Additional file 1 The sequence of the primers**

| **Name** | **Sequence** |
| --- | --- |
| has-miR-25-3p-Forward | 5’-CATTGCACTTGTCTCGGTCTGA-3’ |
| hsa-miR-101-3p-Forward | 5’-TACAGTACTGTGATAACTGAA-3’ |
| hsa-miR-221-3p-Forward | 5’-AGCTACATTGTCTGCTGGGTTTC-3’ |
| hsa-miR-378a-3p-Forward | 5’-ACTGGACTTGGAGTCAGAAGGC-3’ |
| hsa-miR-381-3p-Forward | 5’-TATACAAGGGCAAGCTCTCTGT-3’ |
| hsa-miR-490-3p-Forward | 5’-CAACCTGGAGGACTCCATGCTG-3’ |
| human U6-Forward | 5’-GAAGGATGACACGCAAATTCG-3’ |
| human PLAGL2-Forward | 5’-TCAAGAAGAGCCACTCGCAG-3’ |
| human PLAGL2-Reverse | 5’-CTTGGTCCCCATTACGTCCC-3’ |
| human β-actin-Forward | 5’-CTCACCATGGATGATGATATCGC-3’ |
| human β-actin-Reverse | 5’-AGGAATCCTTCTGACCCATGC-3’ |
