# Supplementary material for: MiR‐378a‐3p as a putative biomarker for hepatocellular carcinoma diagnosis and prognosis: Computational screening with experimental validation
Source: Clin Transl Med. 2021 Feb 14;11(2):e307. doi: 10.1002/ctm2.307 (PMC7882078; doi:10.1002/ctm2.307)
Supplement: Supplementary file 2 — Supporting Information [file CTM2-11-e307-s002.doc]

**Additional file 2 Key microRNAs and network parameters predicted based on HCC-Net1**

| **microRNA** | **Gm** | **NOD** | **TF** | **TFP** |
| --- | --- | --- | --- | --- |
| miR-101-3p | 201 | 57 | 33 | 0.1642 |
| miR-190b | 220 | 52 | 38 | 0.1727 |
| miR-191-5p | 130 | 30 | 23 | 0.1769 |
| miR-196b-5p | 286 | 84 | 41 | 0.1434 |
| miR-214-3p | 104 | 33 | 15 | 0.1442 |
| miR-221-3p | 162 | 33 | 24 | 0.1481 |
| miR-25-3p | 546 | 149 | 93 | 0.1703 |
| miR-324-3p | 76 | 28 | 13 | 0.1711 |
| miR-378a-3p | 176 | 57 | 26 | 0.1477 |
| miR-381-3p | 102 | 37 | 18 | 0.1765 |
| miR-490-3p | 138 | 48 | 20 | 0.1449 |

Note: GM is the number of target genes.
