# Supplementary material for: MiR‐378a‐3p as a putative biomarker for hepatocellular carcinoma diagnosis and prognosis: Computational screening with experimental validation
Source: Clin Transl Med. 2021 Feb 14;11(2):e307. doi: 10.1002/ctm2.307 (PMC7882078; doi:10.1002/ctm2.307)
Supplement: Supplementary file 3 — Supporting Information [file CTM2-11-e307-s003.doc]

**Additional file 3 Key microRNAs and network parameters predicted based on HCC-Net2**

| **microRNA** | **Gm** | **NOD** | **TF** | **TFP** | **microRNA** | **Gm** | **NOD** | **TF** | **TFP** |
| --- | --- | --- | --- | --- | --- | --- | --- | --- | --- |
| let-7g-5p | 186 | 5 | 24 | 0.1290 | miR-381-3p | 35 | 1 | 4 | 0.1143 |
| miR-101-3p | 74 | 2 | 16 | 0.2162 | miR-382-5p | 34 | 3 | 4 | 0.1176 |
| miR-106b-5p | 253 | 1 | 38 | 0.1502 | miR-384 | 17 | 2 | 4 | 0.2353 |
| miR-1250 | 9 | 1 | 1 | 0.1111 | miR-411-5p | 11 | 1 | 2 | 0.1818 |
| miR-125a-3p | 18 | 1 | 2 | 0.1111 | miR-421 | 35 | 2 | 5 | 0.1429 |
| miR-125b-5p | 39 | 1 | 5 | 0.1282 | miR-429 | 55 | 1 | 6 | 0.1091 |
| miR-137 | 47 | 4 | 6 | 0.1277 | miR-448 | 183 | 3 | 21 | 0.1148 |
| miR-138-5p | 31 | 2 | 8 | 0.2581 | miR-483-3p | 29 | 1 | 5 | 0.1724 |
| miR-1-3p | 77 | 3 | 10 | 0.1299 | miR-486-5p | 60 | 3 | 7 | 0.1167 |
| miR-141-3p | 195 | 3 | 21 | 0.1077 | miR-488-3p | 27 | 2 | 5 | 0.1852 |
| miR-144-3p | 54 | 2 | 12 | 0.2222 | miR-490-3p | 56 | 1 | 9 | 0.1607 |
| miR-148b-3p | 59 | 2 | 6 | 0.1074 | miR-491-5p | 80 | 3 | 10 | 0.125 |
| miR-155-5p | 88 | 4 | 12 | 0.1364 | miR-495-3p | 48 | 6 | 6 | 0.125 |
| miR-182-5p | 41 | 4 | 9 | 0.2195 | miR-503-5p | 34 | 2 | 4 | 0.1176 |
| miR-190a-5p | 137 | 2 | 19 | 0.1387 | miR-505-3p | 26 | 1 | 4 | 0.1538 |
| miR-192-5p | 49 | 3 | 6 | 0.1224 | miR-520a-3p | 22 | 1 | 4 | 0.1818 |
| miR-193a-3p | 18 | 1 | 3 | 0.1667 | miR-543 | 45 | 1 | 6 | 0.1333 |
| miR-195-5p | 53 | 1 | 6 | 0.1132 | miR-548b-3p | 39 | 1 | 6 | 0.1538 |
| miR-197-3p | 81 | 1 | 10 | 0.1235 | miR-574-3p | 14 | 1 | 2 | 0.1429 |
| miR-199a-5p | 25 | 1 | 6 | 0.2400 | miR-582-5p | 42 | 3 | 5 | 0.119 |
| miR-19a-3p | 72 | 1 | 8 | 0.1111 | miR-590-3p | 322 | 3 | 42 | 0.1304 |
| miR-203a | 45 | 1 | 7 | 0.1556 | miR-590-5p | 104 | 1 | 15 | 0.1442 |
| miR-203a-3p | 45 | 1 | 7 | 0.1556 | miR-593-3p | 18 | 2 | 3 | 0.1667 |
| miR-204-5p | 158 | 1 | 23 | 0.1456 | miR-595 | 25 | 1 | 4 | 0.16 |
| miR-21-5p | 126 | 6 | 15 | 0.1190 | miR-597 | 30 | 1 | 4 | 0.1333 |
| miR-218-5p | 47 | 1 | 6 | 0.1277 | miR-601 | 16 | 1 | 2 | 0.125 |
| miR-221-3p | 45 | 1 | 7 | 0.1556 | miR-605 | 63 | 2 | 8 | 0.127 |
| miR-222-3p | 33 | 2 | 4 | 0.1212 | miR-613 | 133 | 3 | 20 | 0.1504 |
| miR-22-3p | 110 | 4 | 19 | 0.1727 | miR-615-5p | 27 | 2 | 3 | 0.1111 |
| miR-23b-3p | 219 | 3 | 26 | 0.1187 | miR-621 | 14 | 1 | 6 | 0.4286 |
| miR-24-3p | 44 | 3 | 5 | 0.1136 | miR-626 | 21 | 1 | 4 | 0.1905 |
| miR-25-3p | 183 | 1 | 25 | 0.1366 | miR-627 | 20 | 3 | 4 | 0.2 |
| miR-27b-3p | 240 | 12 | 32 | 0.1333 | miR-637 | 29 | 2 | 6 | 0.2069 |
| miR-299-3p | 19 | 1 | 5 | 0.2632 | miR-653 | 48 | 1 | 7 | 0.1458 |
| miR-29b-3p | 72 | 2 | 9 | 0.125 | miR-671-5p | 20 | 1 | 4 | 0.2 |
| miR-301a-3p | 48 | 2 | 8 | 0.1667 | miR-744-5p | 19 | 3 | 3 | 0.1579 |
| miR-302c-3p | 19 | 1 | 3 | 0.1579 | miR-758-3p | 9 | 1 | 2 | 0.2222 |
| miR-30e-5p | 297 | 6 | 41 | 0.138 | miR-770-5p | 22 | 1 | 3 | 0.1364 |
| miR-31-5p | 44 | 2 | 7 | 0.1591 | miR-876-5p | 21 | 2 | 4 | 0.1905 |
| miR-326 | 95 | 1 | 10 | 0.1053 | miR-885-5p | 32 | 2 | 4 | 0.125 |
| miR-335-5p | 118 | 7 | 14 | 0.1186 | miR-92a-3p | 50 | 2 | 6 | 0.12 |
| miR-338-3p | 37 | 2 | 4 | 0.1081 | miR-934 | 25 | 1 | 4 | 0.16 |
| miR-339-5p | 8 | 2 | 1 | 0.125 | miR-93-5p | 269 | 2 | 41 | 0.1524 |
| miR-33b-5p | 110 | 1 | 13 | 0.1182 | miR-939-5p | 27 | 4 | 3 | 0.1111 |
| miR-34a-5p | 59 | 4 | 14 | 0.2373 | miR-942 | 43 | 1 | 9 | 0.2093 |
| miR-34c-5p | 35 | 2 | 8 | 0.2286 | miR-9-5p | 39 | 1 | 6 | 0.1538 |
| miR-362-3p | 106 | 2 | 15 | 0.1415 | miR-98-5p | 282 | 2 | 42 | 0.1489 |
| miR-378a-3p | 84 | 1 | 10 | 0.119 |  |  |  |  |  |

Note: GM is the number of target genes.
