# Supplementary material for: MiR‐378a‐3p as a putative biomarker for hepatocellular carcinoma diagnosis and prognosis: Computational screening with experimental validation
Source: Clin Transl Med. 2021 Feb 14;11(2):e307. doi: 10.1002/ctm2.307 (PMC7882078; doi:10.1002/ctm2.307)

**Additional file 4 Survival analysis results from TCGA-LIHC datasets. (A)** The relationship between expression level of miR-378a-3p and overall survival of HCC patients. **(B)** The relationship between expression level of miR-378a-3p and TNM stages of HCC patients.


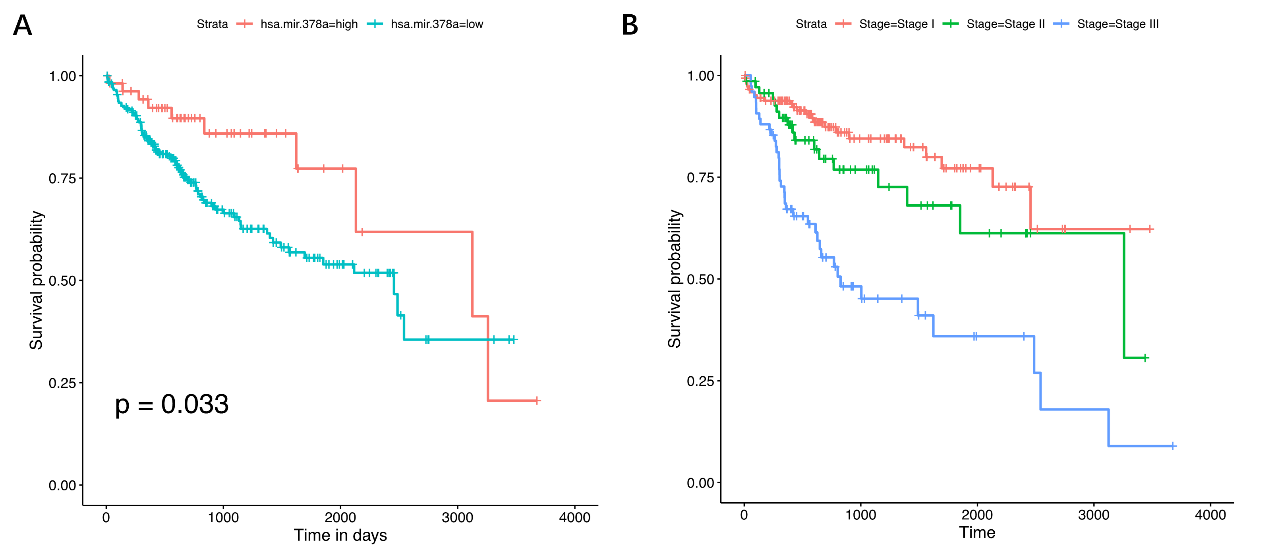

Supplement: Supplementary file 4 — Supporting Information [file CTM2-11-e307-s004.doc]
